# What exercise prescription is optimal to improve body composition and cardiorespiratory fitness in adults living with obesity? A network meta‐analysis

**DOI:** 10.1111/obr.13137

**Published:** 2020-09-08

**Authors:** Grainne O'Donoghue, Catherine Blake, Caitriona Cunningham, Olive Lennon, Carla Perrotta

**Affiliations:** ^1^ School of Public Health, Physiotherapy and Sports Science University College Dublin Dublin Ireland

**Keywords:** exercise training, F.I.T.T, network meta‐analysis, obesity

## Abstract

Current international guidelines recommend people living with obesity should be prescribed a minimum of 300 min of moderately intense activity per week for weight loss. However, the most efficacious exercise prescription to improve anthropometry, cardiorespiratory fitness (CRF) and metabolic health in this population remains unknown. Thus, this network meta‐analysis was conducted to assess and rank comparative efficacy of different exercise interventions on anthropometry, CRF and other metabolic risk factors. Five electronic databases were searched for randomized controlled trials (RCTs) that compared different exercise modalities to improve anthropometry, CRF and/or metabolic health in adults living with obesity. RCTs were evaluated using the Cochrane risk of bias tool. A random effects network meta‐analysis was performed within a frequentist framework. Of the 6663 articles retrieved, 45 studies with a total 3566 participants were included. Results reveal that while any type of exercise intervention is more effective than control, weight loss induced is modest. Interventions that combine high‐intensity aerobic and high‐load resistance training exert beneficial effects that are superior to any other exercise modality at decreasing abdominal adiposity, improving lean body mass and increasing CRF. Clinicians should consider this evidence when prescribing exercise for adults living with obesity, to ensure optimal effectiveness.

**Abbreviations**
BMI: body mass index
BP: blood pressure
CRF: cardiorespiratory fitness
CVD: cardiovascular disease
F.I.T.T.: frequency, intensity, time and type
FBG: fasting blood glucose
HDL: high‐density lipoprotein
HIIT: high‐intensity interval training
NMA: network meta‐analysis
RCT: randomized controlled trial
T2DM: type 2 diabetes mellitus
TG: triglycerides
VO_2max_: maximal oxygen uptake
WC: waist circumference

## 1 BACKGROUND

The prevalence of obesity has tripled over the past 35 years,^1^ and it is estimated that it will affect over one billion people worldwide by 2030.^2^, ^3^, ^4^ Obesity has far reaching negative effects on health, significantly increasing the risk of cardiovascular disease (CVD), metabolic disease and certain cancers,^1^ primarily driven by comorbidities such as type 2 diabetes mellitus (T2DM), dyslipidaemia and hypertension.^2^

Diet, exercise and behaviour modification remain the cornerstones of obesity management.^1^ However, sole focus on weight loss is not the optimal, as it fails to consider cardiorespiratory fitness (CRF), which can, at medium to high fitness levels, attenuate the adverse consequences of obesity on health, irrespective of body mass index (BMI).^5^, ^6^, ^7^ Growing evidence suggests that improved CRF largely neutralizes the adverse effects of increased adiposity as well as other traditional CVD risk factors.^8^

Current guidelines recommend that exercise programmes for weight loss in obesity prioritize continuous moderate intensity aerobic exercise, and supplement this approach, where possible, with resistance training.^9^, ^10^ However, multiple exercise modalities, with varying intensities, feature in the obesity literature and data identifying the relative effect of the different exercise interventions on CRF, body composition and metabolic health are somewhat inconsistent.^11^ Several randomized controlled trials (RCTs) and systematic reviews report improvements in maximal oxygen uptake (VO_2max)_, waist circumference (WC) and BMI with aerobic and/or combined training,^11^, ^12^, ^13^, ^14^, ^15^, ^16^, ^17^, ^18^, ^19^ whereas improvements in VO_2max_ and in some cases decreased BMI and blood pressure are similarly reported by applying resistance training alone.^16^, ^18^, ^19^, ^20^, ^21^, ^22^, ^23^

It is difficult in this context to determine the superiority of the different exercise interventions using only individual RCTs or even pairwise meta‐analysis, as often these studies were designed to compare one or more exercise interventions with data from nonexercising control groups and therefore cannot discriminate between exercise modalities, making it difficult to draw firm conclusions.

Also called mixed treatments comparison or multiple treatments comparison meta‐analysis, network meta‐analysis (NMA) expands the scope of a conventional pairwise analysis by analysing simultaneously both the direct and the indirect evidence from different studies, allowing for estimation of the relative effectiveness among all interventions and rank ordering of the interventions, even where two interventions comparisons are lacking.^24^

To date, no systematic review has pooled the effects of different training modalities on outcomes of anthropometry, CRF and cardiometabolic risk factors, focusing exclusively on adults living with obesity and including only RCTs where exercise is the only intervention being investigated. Therefore, this study aimed to conduct a NMA of RCTs to (i) assess the comparative efficacy of different exercise types and their intensities on anthropometry, fitness and other metabolic markers in adults living with obesity and (ii) establish a hierarchy of these exercise interventions.

## 2 METHODS AND ANALYSIS

### 2.1 Registration

This systematic review and NMA is reported in accordance with the Preferred Reporting Items for Systematic Reviews and Meta‐Analyses (PRISMA) statement.^25^ The study protocol was registered (registration number: CRD4201811373) with the International Prospective Register of Systematic Reviews (PROSPERO).

### 2.2 Search strategy

Five electronic databases (PubMed, EMBASE, Cochrane Central Register of Controlled Trials [CENTRAL], Cumulative Index to Nursing and Allied Health Literature [CINAHL] and Sport Discus) were searched. The search strategy was constructed around the PICOS tool: (P) Population: adults living with obesity; (I) Intervention: exercise; (C) Comparator: other exercise modality or no exercise control; (O) Outcomes: anthropometry, CRF and cardiovascular risk factors and (S) Study type: RCTs. A complete list of the search terms is available in the additional materials section (Table S1). In addition to the databases, the reference lists of included articles were scanned for articles that met the inclusion criteria.

### 2.3 Eligibility criteria

RCTs published in scientific peer reviewed papers, written in English and from the past 30 years (January 1998 to November 2019) were included (conference abstracts, reports and theses were excluded). Adult‐based studies were defined by a study population aged between ≥18 years and <65 years and obesity was defined as a BMI > 30 kg m^−2^ or body fat > 30% in women and >25% in men. Articles where the population was reported to be taking medication directed at excess weight or had a diagnosed pathology (e.g., type 2 diabetes) were excluded. Outcomes of interest included anthropometry (body weight [BW], BMI, percentage body fat [%BF], WC), CRF as measured by maximal oxygen uptake (VO_2max_) and risk factors associated with the metabolic syndrome (systolic and diastolic blood pressure [BP], total high‐density lipoprotein [HDL] cholesterol, triglycerides (TG) and fasting blood glucose [FBG]).

### 2.4 Exercise categories

Seven categories were used to classify the exercise interventions for the included RCTs:

1. Aerobic exercise; vigorous intensity (AE‐V)
2. Aerobic exercise; moderate intensity (AE‐M)
3. Resistance training; high load (R‐HI)
4. Resistance training; low‐to‐moderate load (R‐LM)
5. Combined high; vigorous intensity aerobic/high‐load resistance (COM‐HI)
6. Combined low‐moderate; moderate intensity aerobic/low‐to‐moderate load resistance (COM‐LM)
7. No exercise (control group)

Each category was devised using the frequency, intensity, time and type (F.I.T.T.) principle of exercise prescription and the ACSM's estimates of cardiorespiratory and resistance exercise intensity.^26^ Vigorous aerobic exercise intensity was defined as >65% VO_2max_ or >65% HRR or >75% HR_max_ and moderate as 45–65% VO_2max_ or 50–65% HRR or >65–75% HR_max_. High‐load resistance training was defined as >75% of the one repetition max (1RM) and moderate as 50–75% 1RM. A detailed definition of each exercise category is provided in Table 1.

**TABLE 1:** Definition of the exercise training interventions using the F.I.T.T. principle

Type of exercise	Abbreviation	Definition
Aerobic; vigorous intensity	AE‐V	Frequency: 3–5 times per week, each session lasting 30–60 min
		Intensity: >65% VO_2max_ or >65% HRR or >75% HR_max_
		Time: ≥8 weeks
		Type: Any mode of aerobic only (e.g., walking, running, cycling and swimming)
Aerobic; moderate intensity	AE‐M	Frequency: 3–5 times per week, each session lasting 30–60 min
		Intensity: 45–65% VO_2max_ or >50–65% HRR or 65–75% HR_max_
		Time: ≥8 weeks
		Type: Any mode of aerobic only (e.g., walking, running, cycling and swimming)
Resistance; high load	R‐HI	Frequency: 3 times per week; each session lasting 30–60 min
		Intensity: Average maximum load > 75% 1RM
		Time: ≥8 weeks
		Type: Any mode of resistance training (e.g., free weights, weights machines and resistance bands)
Resistance; low‐moderate load	R‐LM	Frequency: 3 times per week; each session lasting 30–60 min
		Intensity: Average maximum load 50–75% 1RM
		Time: ≥8 weeks
		Type: Any mode of resistance training (e.g., free weights, weights machines and resistance bands)
Combined; high intensity	COM‐HI	A combination of aerobic; vigorous intensity and resistance; high load
Combined; low‐moderate intensity	COM‐LM	A combination of aerobic; moderate intensity and resistance; low‐moderate intensity
Control	CON	No exercise

Abbreviations: F.I.T.T., frequency, intensity, time and type; HR_max_, maximum heart rate; HRR, heart rate reserve; RM, repetition maximum; VO_2max_, maximal oxygen uptake.

### 2.5 Study selection

Endnote X8 literature management software was used to manage the literature search records. The selection process consisted of three phases. In the initial phase, three reviewers independently screened the yielded articles based on title. In the case of doubt, the articles were included in the abstract review phase. In Phase 2, all articles selected from the initial phase were reviewed by abstract and assessed for eligibility by two independent reviewers. Any disagreements were resolved by discussion between reviewers and consultation with a third party from the review team. In the final phase, the remaining articles were fully reviewed by the same two independent reviewers that reviewed abstracts, using the predetermined inclusion criteria. Any disagreements between reviewers in this phase were again resolved by discussion within the wider team.

### 2.6 Data extraction

A nine‐item, standardized and prepiloted data extraction form was used to record data from the included studies under the following headings: (i) author, (ii) year of publication, (iii) country, (iv) study period, (v) sample size, (vi) mean age, (vii) mean baseline, (viii) follow‐up BW, BMI, %BF, WC, BP, HDL, TG, FBG and VO_2max_ and (ix) details of the exercise intervention. Data were recorded for each exercise intervention using the F.I.T.T.

### 2.7 Risk of bias of individual studies

Two authors (G.O. and C.C.) independently assessed the risk of bias (ROB), in accordance with the Cochrane Handbook version 5.1.0 tool for assessing ROB in RCTs.^27^ The following seven domains were considered: (i) randomized sequence generation, (ii) treatment allocation concealment, blinding of (iii) participants and (iv) personnel, (v) incomplete outcome data, (vi) selective reporting and (vii) other sources of bias. Trials were categorized into three levels of ROB by the number of components for which high ROB potentially existed: high risk (five or more), moderate risk (three or four) and low risk (two or less). All studies would, by default, be classified as high ROB with respect to the category ‘blinding of participants’ given it was impossible to blind participants to group assignment in exercise intervention protocols. Therefore, this component was not included in the overall ROB score.

### 2.8 Data analysis

First, we qualitatively described included trials, their exercise intervention characteristics and their relative contributions to the overall body of evidence available. We converted outcomes to standard units and calculated mean difference, subtracting the mean at the end of the intervention versus baseline. End of the intervention was always at the end of exercise participation to avoid any potential wash out. If the standard deviation (SD) difference was missing, we calculated using the SD formula of the difference between two means using the following formula: $\;\sqrt[{2}]{\frac{{s_{1}}^{2}}{n_{1}}+\frac{{s_{2}}^{2}}{n_{2.}}}$.

Omitted data (e.g., missing SDs or only *p* values reported), was dealt with by using the *metaeff* command procedures in Stata to calculate SMDs and 95% confidence intervals (CIs) from available data.^28^ When a study had multiple intervention arms and where we defined two arms as constituting the same exercise intervention (e.g., 30 min of continuous moderately intense aerobic exercise versus 3 × 10‐min bouts of moderately intense aerobic exercise), the data from the intervention groups were pooled. Where studies used the same outcome measure, we pooled mean, SD and sample size under each of the predefined exercise categories. Regarding VO_2max_, when it was reported in relative terms (ml kg^−1^ min^−1^), absolute VO_2max_ was calculated using the mean BWs for the appropriate time points. For each exercise intervention employed in a study, the absolute change in VO_2max_ (L min^−1^) was calculated, whereas the pooled SD (SD_P_) was calculated using the sample size (*n*) and SDs from before and after in each study or study group.^29^

As interventions are by definition heterogeneous and pairwise meta‐analytic estimates are usually reported in addition to the network estimates,^30^ random effects pairwise meta‐analyses were first used to obtain pooled SMDs (with associated CIs) for weight loss, BMI, WC, %BF and fitness, respectively, with the *I* ^2^ statistic used to quantify heterogeneity. Transitivity is a key assumption of NMA and refers to the belief that indirect comparison is a valid estimate of the unobserved direct comparison^31^ and that all studies have homogenous distribution of effect modifiers.^32^ Because duration of the exercise intervention (number of weeks) had been signalled as a potential modifier for all outcomes, we explored by pairwise meta‐analyses with duration as a covariate as to whether it modified the magnitude and direction of the estimates. As the intervention duration did not modify the estimates for each of the outcomes (Table S2), we proceeded to NMA.

We used STATA software (Version 15.1) command ‘mvmeta’ to perform a multivariate NMA within a frequentist framework^33^ in accordance with current PRISMA NMA guidelines.^25^ To allow for between‐study heterogeneity, a random effects NMA was performed to calculate pooled estimates and 95% CIs. The mean difference was used as the effect estimate to analyse the results.

In addition to the pairwise meta‐analysis, transitivity assumptions were also assessed through assessment of individual studies inclusion criteria, whether all participants in the network could have been randomized to any intervention, logical inference and using consistency models.^34^ Consistency, whereby the treatment effect estimated from direct comparisons are consistent with those estimated from indirect comparisons, was tested through the Wald test and node splitting method.

The ‘networkplot’ function of STATA was employed to create network plots that describe and present the geometry of the different exercise interventions. In the plots generated, nodes represent the different exercise interventions and the control condition of no exercise and lines connecting the nodes represented the direct head‐to‐head comparisons between interventions. The size of each node and the thickness of each line connecting the nodes are proportional to the number of studies.

Intervention hierarchy was summarized and reported as a *P* score.^35^ The *P* score is considered as a frequentist analogue to surface under the cumulative ranking curve (SUCRA) values and measures the extent of certainty that a treatment is better than another treatment, averaged over all competing treatments. The *P* score ranges from 0 to 1, where 1 indicates best treatment with no uncertainty and 0 indicates worst treatment with no uncertainty. While the *P* score or SUCRA can be usefully re‐expressed as the percentage of effectiveness or acceptability of the exercise interventions, such scores should be interpreted cautiously unless there are actual clinically meaningful differences between interventions.^36^ To check for the presence of bias due to small‐scale studies, which may lead to publication bias in NMA, a network funnel plot was generated (Figure S8) and visually inspected using the criterion of symmetry.^37^

## 3 RESULTS

### 3.1 Literature selection

**FIGURE 1:**
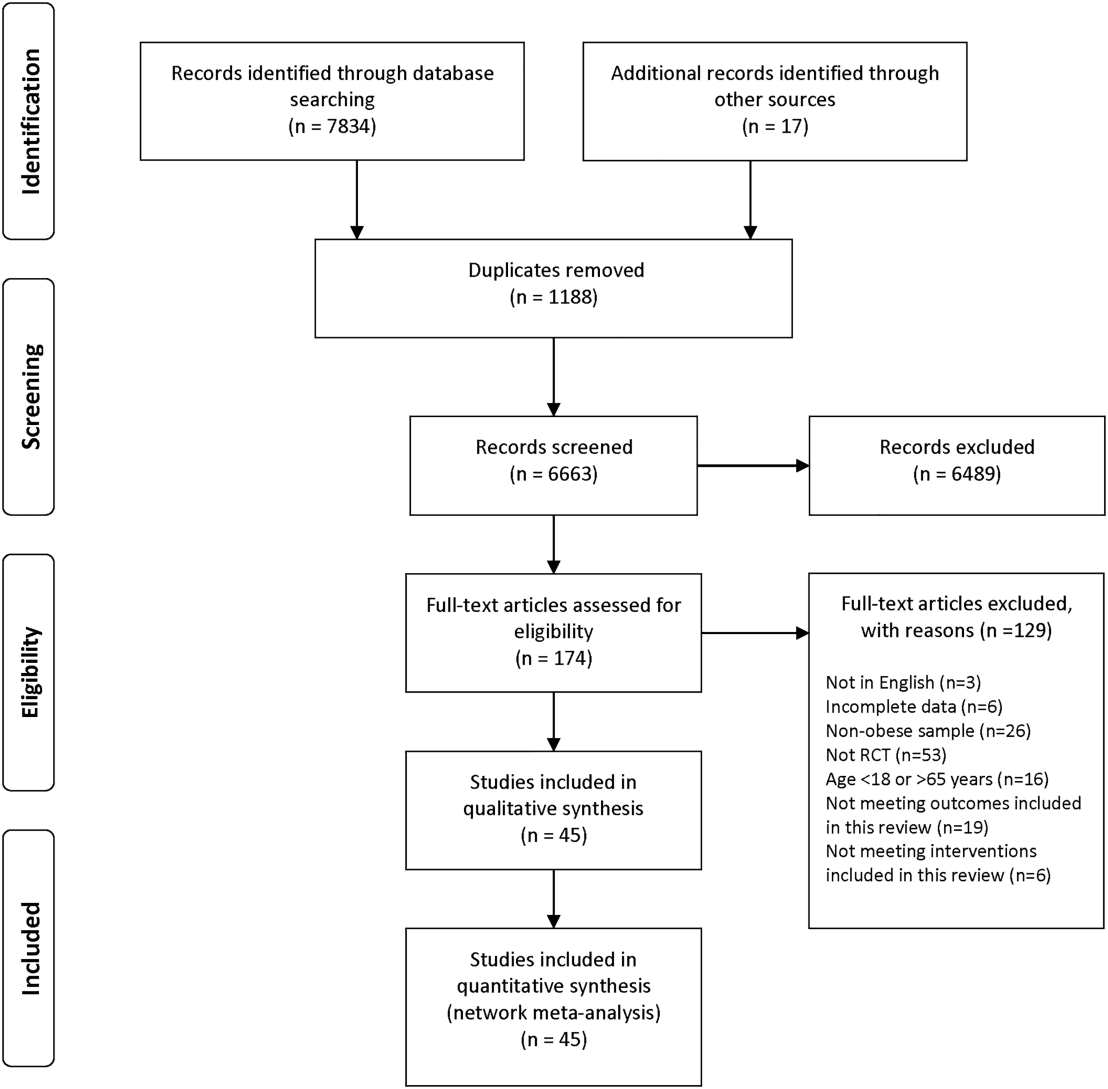
PRISMA flow chart

A total of 6663 studies were initially identified. Following review by title and abstract, 174 studies progressed to full manuscript review. Of these, 129 were excluded as they did not fulfil our inclusion criteria. The remaining 45 studies were included in this review.^13^, ^14^, ^15^, ^17^, ^21^, ^38^, ^39^, ^40^, ^41^, ^42^, ^43^, ^44^, ^45^, ^46^, ^47^, ^48^, ^49^, ^50^, ^51^, ^52^, ^53^, ^54^, ^55^, ^56^, ^57^, ^58^, ^59^, ^60^, ^61^, ^62^, ^63^, ^64^, ^65^, ^66^, ^67^, ^68^, ^69^, ^70^, ^71^, ^72^, ^73^, ^74^, ^75^, ^76^, ^77^ The detailed process is illustrated in Figure 1.

### 3.2 Characteristics of the included studies

The characteristics of the included studies are presented in Table 2. The studies were conducted in North America (*n* = 16), * Europe (*n* = 11), † Asia (*n* = 9), ‡ Australia (*n* = 5),^14^, ^15^, ^48^, ^59^, ^60^ South America (*n* = 3)^39^, ^42^, ^66^ and Africa (n = 1).^62^ A total of 3566 people with an average age of 43.3 (SD: 13.1) years, were investigated across the 45 studies. Of these, 2696 (76%) were female. Eighteen studies were women only, § eight were men only ¶ and the remaining 19 included both men and women. Reported baseline demographics included mean BW (90.5 kg [SD: 8.5]), BMI (32.3 kg m^−2^ [SD: 3.3]), WC (105 cm [SD: 9.3]), %BF (37.9% [SD: 6.8]) and CRF (2.46 L min^−1^ [0.6]).

**TABLE 2:** Summary of studies included in network meta‐analysis indicating the exercise intervention used and the outcome measures reported for comparison within the analysis

Study	Country	Duration (weeks)	Sample N/F/M	Mean age (SD)	Exercise category	Summary description of exercise intervention Frequency, intensity, time and type (F.I.T.T.)	Outcome measures reported
Alizadeh et al.^38^	Iran	12	15/15/0 15/15/0 15/15/0	34.5 (6.2) 33.1 (7.7) 32.4 (9.5)	CON AE‐M AE‐M	No exercise Continuous walking; 40 min; 65–75% HR_max_; 5 days week^−1^ Intermittent walking; 3 bouts summing up to 40 min; 65–75% HR_max_; 5 days week^−1^	Weight, BMI
Alves et al.^39^	Brazil	26	78/78/0 78/78/0	37 (10.6) 39.4 (11.1)	CON AE‐M	No exercise Continuous walking; 40 min; 40–60% HRR; 3 days week^−1^	Weight, BMI
Arad et al.^40^	USA	12	14/14/0 14/14/0	30.2 (7) 29.1 (4)	CON AE‐V	No exercise HITT: 6 min; 50% HRR, 4 intervals (60 s at 80–90% HRR) and 4 recovery minutes (210 s 50% HRR); 24 min; 3 days week^−1^	Weight, BMI, %fat, fitness
Arslan^41^	Turkey	8	20/20/0 29/29/0	37 (9.1) 41.6 (6.7)	CON AE‐V	No exercise Weeks 1–4: 60–70% HR_max_; 40 min; 3 days week^−1^ and then Weeks 5–8: 80% HR_max_; 50 min; 3 days week^−1^ (step‐aerobics)	Weight, BMI, %fat
Bonfante et al.^42^	Brazil	26	10/0/10 12/0/12	49.1 (6.2) 49.1 (5.5)	CON COM‐LM	No exercise 60‐min session; resistance (3 sets of 8–10 max reps) and endurance (walking/running) 60–65% VO_2_ peak; 3 days week^−1^	Weight, BMI, WC, %fat, fitness, HDL, TG, FBG
Borg et al.^43^	Finland	26	27/0/27 28/0/28 29/0/29	35–50^d^	CON AE‐M R‐LM	No exercise 60–70% VO_2max_, 45 min; 3 days week^−1^ 60–75% 1RM, 8 reps/3 sets/6 exercises, 45 min; 3 days week^−1^	Weight, WC
Chih‐Hui et al.^44^	Taiwan	12	12/3/9 12/4/8 12/3/9 12/4/8	20.8 (0.7) 21.8 (0.7) 20.9 (0.4) 20.7 (0.6)	CON AE‐M AE‐M AE‐V	No exercise 40–50% HRR; 60 min; 3 days week^−1^ 60–70% HHR; 60 min; 3 days week^−1^ 70–80% HHR; 60 min; 3 days week^−1^	Weight, BMI, WC
Church et al.^45^	USA	26	96/96/0 145/145/0 90/90/0 96/96/0	57.2 (5.9) 57.9 (6.5) 56.7 (6.4) 56.4 (6.3)	CON AE‐M AE‐M AE‐M	No exercise 73 min week^−1^; 50% VO_2_ peak treadmill/cycle ergometer 139 min week^−1^; 50% VO_2_ peak treadmill/cycle ergometer 192 min week^−1^; 50% VO_2_ peak treadmill/cycle ergometer	Weight, WC, %fat, fitness, HDL, TG, FBG
Croymans et al.^46^	USA	12	8/8/0 28/28/0	22.0 (1.5) 21.5 (2.1)	CON R‐HI	No exercise 3 days week^−1^; 60 min; 8 global exercises; 2 sets (15 reps); progressing from 100% 15 rep max to 100% 6 rep max	Weight, BMI, WC, %fat, BP
Dengel et al.^47^	USA	52	9/0/9 10/0/10	62.8 (2.5) 60.3 (2.4)	CON AE‐V	No exercise 60% HRR; 30 min; 3 days week^−1^ progress to 85% HRR; 40 min	Weight, %fat, fitness, FBG
Donges et al.^48^	Australia	12	11/0/11 12/0/12 12/0/12 12/0/12	49.5 (2.6) 45.4 (1.7) 51.7 (2.1) 46.2 (1.4)	CON AE‐V R‐HI COM‐HI	No exercise 75–80% HR_max_; 40–60 min; 3 days week^−1^; cycle ergometer 75–80% 1RM; 10 exercises; 3–4 sets; 10 reps; 3 days week^−1^ 50% aerobic as above and 50% resistance as above; 3 days week^−1^	Weight, %fat, fitness
Donnelly et al.^49^ ^,^ ^a^	USA	42	15/15/0 31/31/0 32/32/0	21.8 (2.6) 22.6 (2.9) 22.6 (3.2)	CON AE‐V AE‐V	No exercise 70–80% HR_max_; 48 min; 5 days week^−1^; treadmill 70–80% HR_max_; 63 min; 5 days week^−1^; treadmill	Weight, BMI, %fat, fitness
Donnelly et al.^49^ ^,^ ^a^	USA	42	11/0/11 22/0/22 30/0/30	23.3 (3.4) 23.5 (3.2) 23.3 (3.7)	CON AE‐V AE‐V	No exercise 70–80% HR_max_; 31 min; 5 days week^−1^; treadmill 70–80% HR_max_; 42 min; 5 days week^−1^; treadmill	Weight, BMI, %fat, fitness
Foster‐Schubert et al.^50^	USA	52	87/87/0 117/117/0	57.4 (4.4) 58.1 (5)	CON AE‐V	No exercise 70–85% HR_max_; 45 min; 5 days week^−1^; treadmill or bike	Weight, BMI, WC, %fat, fitness
Frank et al.^51^	USA	52	86/86/0 87/87/0	60.6 (6.6) 60.7 (6.7)	CON AE‐M	No exercise 60–75% HRR; 45 min; 5 days week^−1^	BMI, %fat, TG, FBG
Gram et al.^52^	Denmark	26	18/9/9 35/19/16 39/21/18 38/20/18	35 35 33 36	CON AE‐M AE‐M AE‐V	No exercise 320 kcal (f)/425 kcal (m); 5 days week^−1^, no intensity prescribed 320 kcal (f)/425 kcal (m); 5 days week^−1^; 50% VO_2_ peak 320 kcal (f)/425 kcal (m); 5 days week^−1^; 70% VO_2_ peak	BMI, fitness, HDL, TG
Herring et al.^53^	UK	12	10/−/− 11/−/− 12/−/−	24–68^d^	CON R‐LM AE‐M	No exercise 60% 1RM; global exercises; 45–60 min; 3 days week^−1^ 50–70% HR_max_: 45–60 min; 3 days week^−1^	BMI, WC, fitness
Hintze et al.^54^	Canada	52	34/24/0 36/36/0	29 25	CON R‐HI	No exercise 70–80% 1RM; global; 45 min; 3 days week^−1^	Weight, BMI
Ho et al.^15^ ^,^ ^b^	Australia	12	21/−/− 25/−/− 26/−/− 25/−/−	52 (1.8) 55 (1.2) 52 (1.1) 53 (1.3)	CON AE‐M R‐ML COM‐ML	No exercise 60% HRR; 30 min; 5 days week^−1^; walking 75% 1RM; 5 global exercises × 4 sets, 12 reps 60% HRR; 15 min; 5 days week^−1^ and 75% 1RM; 5 global exercises × 2 sets, 12 reps	Weight, BMI, WC, %fat, HDL, TG, FBG
Ho et al.^14^ ^,^ ^b^	Australia	12	21/−/− 25/−/− 26/−/− 25/−/−	52 (1.8) 55 (1.2) 52 (1.1) 53 (1.3)	CON AE‐M R‐ML COM‐ML	No exercise 60% HRR; 30 min; 5 days week^−1^; walking 75% 1RM; 5 global exercises × 4 sets, 12 reps 60% HRR; 15 min; 5 days week^−1^ and 75% 1RM; 5 global exercises × 2 sets, 12 reps	BP
Irving et al.^55^	USA	16	9/9/0 15/15/0 12/12/0	51 (9) 51 (9) 51 (9)	CON AE‐M AE‐V	No exercise RPE 10–12; expenditure 400 kcal; 5 days week^−1^ RPE 15–17; expenditure 400 kcal; 5 days week^−1^	Weight, BMI, WC, %fat, fitness, BP, HDL, TG, FBG
Irwin et al.^56^	USA	52	86/86/0 85/85/0	60.5 (6.7) 60.6 (6.6)	CON AE‐M	No exercise 60–75% HRR; 45 min; 5 days week^−1^	Weight, BMI, %fat
Jabbour^57^ ^,^ ^c^	Canada	8	12/7/5 12/7/5	23.1 (3.3) 22.5 (2.3)	CON AE‐V	No exercise ~350% of VO_2max_ × 6 s, followed by 2‐min recovery × 6 times; total duration = 15 min; 3 days week^−1^; cycle ergometer	Weight, BMI, fitness
Jabbour et al.^58^ ^,^ ^c^	Canada	8	12/7/5 12/7/5	23.1 (3.3) 23.3 (3.3)	CON AE‐V	No exercise ~350% of VO_2max_ × 6 s, followed by 2‐min recovery × 6 times; total duration = 15 min; 3 days week^−1^; cycle ergometer	Weight, BMI
Keating et al.^59^	Australia	8	12/9/3 12/6/6 12/7/5 12/9/3	39.1 (2.9) 44.2 (2.8) 45.5 (2.3) 45.6 (3.6)	CON AE‐V AE‐M AE‐M	No exercise 70% VO_2_ peak; 45 min; 3 days week^−1^ 50% VO_2_ peak; 60 min; 4 days week^−1^ 50% VO_2_ peak; 45 min; 3 days week^−1^	Weight, BMI, WC, fitness, BP, HDL, TG, FBG
Keating et al.^60^	Australia	8	14/12/2 15/13/2	44.2 (2.8) 45.4 (1.9)	CON R‐HI	No exercise 80–85% 1RM; global exercises; 45–60 min; 3 days week^−1^	Weight, BMI, WC, fitness, BP, HDL, TG, FBG
Kline et al.^61^	USA	12	16/8/9 27/12/15	45.9 (2.2) 47.6 (1.3)	CON COM‐LM	No exercise 60% HHR × 40 min; 4 days week^−1^ and 8 global exercises; 2 sets × 12 reps; 2 days week^−1^	Weight, WC, %fat
Mengistie et al.^62^	Ethiopia	12	35/6/29 35/5/30	45.3 (5.4) 46.2 (5.5)	CON COM‐LM	No exercise 60–80% HR_max_; 20 min; 4 days week^−1^; treadmill or bike and 70% 1RM; 7 exercise (2–3 sets, 12 reps); 20 min	Weight, BMI, WC, %fat, fitness, BP, FBG
Moghasdasi et al.^63^	Iran	12	8/0/8 8/0/8	41.2 (6.1) 41.2 (6.1)	CON AE‐V	No exercise Treadmill; 75–80% VO_2_ peak; 45 min; 4 days week^−1^	Weight, BMI, %fat, fitness, FBG
Mohanka et al.^64^	USA	52	86/86/0 85/85/0	60.5 (6.7) 60.6 (6.6)	CON AE‐M	No exercise 60–75% HRR; 45 min; 5 days week^−1^	Weight, HDL, TG, fitness
Mora‐Rodriguez et al.^65^	Spain	26	20/−/− 20/−/−	54 (9) 54 (9)	CON AE‐V	No exercise HITT (90% HR_max_ × 4 min, 70% HR_max_ × 3 min) × 45 min; 3 days week^−1^	Weight, BMI, WC, %fat, fitness, HDL, TG
Nunes et al.^66^	Brazil	16	13/13/0 13/13/0 12/12/0	54–65.5^d^	CON R‐ML R‐ML	No exercise 70% 1RM; global; 3 sets/8–12 reps; 3 days week^−1^ × 16 weeks 70% 1RM; global; 6 sets/8–12 reps; 3 days week^−1^ × 16 weeks	BMI, WC, %fat, HDL, TG
Quist et al.^67^	Denmark	26	18/9/9 35/19/16 39/20/19 38/20/18	35 (7) 35 (7) 33 (7) 27 (7)	CON AE‐M AE‐M AE‐V	No exercise Commuting to and from work; 45 min; 5 days 50% VO_2max_; 55 min; 5 days week^−1^; treadmill/cycle ergometer 70% VO_2max_; 37 min; 5 days week^−1^; treadmill/cycle ergometer	Weight, BMI, fitness
Ross et al.^68^	Canada	26	70/49/26 73/49/24 76/49/27 76/49/27	52.2 (8.2) 52.1 (7.4) 50.9 (8.6) 50.3 (8.1)	CON AE‐M AE‐M AE‐V	No exercise 50% VO_2_ peak; 31 min; 5 days week^−1^; treadmill 50% VO_2_ peak; 58 min; 5 days week^−1^; treadmill 75% VO_2_ peak; 40 min; 5 days week^−1^; treadmill	Weight, BMI, WC, %fat, fitness, BP, HDL, TG, FBG
Rustaden et al.^69^	Norway	12	36/36/0 37/37/0 35/35/0 35/35/0	40 (10) 30 (10) 38 (9) 42 (11)	CON R‐HI R‐HI R‐HI	No exercise Body pump; 800 reps between 10 exercises; 60 min; 3 days week^−1^ 80–90% 1RM; 8 global exercises; 45 min; 3 days week^−1^ 80–90% 1RM; 8 global exercises; 45 min; 3 days week^−1^	Weight, BMI, %fat
Sarsan et al.^21^	Turkey	12	24/24/0 26/26/0 26/26/0	43.6 (6.46) 41.7 (7.62) 42.5 (10.7)	CON AE‐M R‐ML	No exercise 50–80% HRR; 30–45 min; 5 days week^−1^; treadmill or bike 40–80% 1RM; 6 global exercises; 30–45 min; 3 days week^−1^	Weight, BMI, WC, fitness, BP
Schroeder et al.^17^	USA	8	17/11/6 17/10/7 17/10/7 18/11/7	58 (6) 58 (7) 57 (9) 58 (7)	CON AE‐V R‐HI COM‐HI	No exercise 85% HR_max_; 60 min; 3 days week^−1^; treadmill or bike 12 global exercises; 3 sets 10–14 maximal repetitions 30 min of aerobic and 30‐min resistance programme	Weight, BMI, WC, %fat, fitness, BP, HDL, TG, FBG
Sheikholeslami‐Vatani et al.^70^	Iran	8	10/0/10 10/0/10 10/0/10	23.2 (1.4) 23.2 (1.4) 23.2 (1.4)	CON COM‐HI COM‐HI	No exercise 80% HR_max_; 10 min and 80% 1RM; 6 global exercises; 3 days week^−1^ 80% HR_max_; 10 min and 80% 1RM; 6 global exercises; 3 days week^−1^	Weight, BMI, %fat
Soori et al.^71^	Iran	8	8/8/0 8/8/0 8/8/0 8/8/0	45–60^d^	CON AE‐M R‐ML COM‐ML	No exercise 40–75% HR_max_; 45 min; 3 days week^−1^; swimming/water walking 40–60% 1RM; 6 global exercises × 3 sets (12 reps); 3 days week^−1^ 22‐min aerobic (AE‐M); 22‐min resistance (R‐ML) programmes	Weight, BMI, %fat, HDL, TG
Stensvold et al.^13^	Norway	12	11/−/− 11/−/− 11/−/− 10/−/−	47.3 (10.2) 49.9 (10.1) 52.9 (10.4) 50.9 (7.6)	CON AE‐V R‐HI COM‐HI	No exercise 90–95% HRR; 4 min; 3‐min recovery; 4 times (45 min); 3 days week^−1^; treadmill 80% 1RM; 3 sets, 8–12 reps; 5 global exercises; 45 min; 3 days week^−1^ 90–95% HRR; 4 min; 3‐min recovery; 4 times (45 min); 1 day week^−1^; treadmill and 80% 1RM; 3 sets, 8–12 reps; 5 global exercises; 45 min; 2 days week^−1^	Weight, BMI, WC, fitness, BP, HDL, TG, FBG
Tjonne et al.^72^	Norway	16	10/5/5 10/5/5 12/5/7	49.6 (9) 52 (10.6) 55.3 (13.2)	CON AE‐M AE‐V	No exercise 70% HR_max_; 45 min; 3 days week^−1^; walking/running; treadmill 90% HR_max_; 4 × 4‐min interval with 3 × 4‐min recovery	Weight, BMI, WC, fitness, BP, HDL
Tong et al.^73^	China	12	18/18/0 18/18/0 18/18/0	21.3 (1) 21.3 (1) 20.7 (1.5)	CON AE‐V AE‐V	No exercise 80 × 6 s all‐out cycle sprint with 9 s passive recovery; 4 days week^−1^ 90% VO_2max_ × 4 min; 3‐min recovery (until 400 kJ achieved); 4 days week^−1^	Weight, BMI, %fat, fitness
Tseng et al.^74^	Taiwan	12	10/0/10 10/0/10 10/0/10 10/0/10	22 (0.7) 22.2 (1.1) 21.3 (0.6) 22.3 (1)	CON AE‐M R‐ML COM‐ML	No exercise 50–70% HR_max_; 60 min; 5 days week^−1^ 50–80% 1RM; 8 global exercises 3 sets × 12 reps; 5 days week^−1^ Aerobic (AE‐M) × 3 days and resistance (R‐ML) × 2 days	Weight, BMI, WC, BP, TG, FBG
Tumiati et al.^75^	Italy	38	25/21/4 27/14/13	51 (11) 48 (12)	CON COM‐LM	No exercise 10% below maximal intensity reached in the 2kmWT; 50 min; 1 day week^−1^ and global resistance exercises; 2 days week^−1^	Weight, BMI, WC, fitness
van Aggel‐Leijssen et al.^76^	Netherlands	12	8/8/0 7/7/0 6/6/0	42.5 (6.4) 32.8 (9.6) 37.7 (6.4)	CON AE‐M AE‐M	No exercise 40% VO_2max_; 57 (6) min (250 kcal); 3 days week^−1^; cycle ergometer 40% VO_2max_; 57 (6) min (250 kcal); 3 days week^−1^; cycle ergometer	Weight, BMI, %fat, fitness
van Aggel‐Leijssen et al.^77^	Netherlands	12	8/0/8 8/0/8 8/0/8	43.3 (5.4) 43.4 (6.3) 40 (6.3)	CON AE‐M AE‐V	No exercise 40% VO_2max_; 57 (8) min (350 kcal); 3 days week^−1^; cycle ergometer 70% VO_2max_; 33 (2) min (350 kcal); 3 days week^−1^; cycle ergometer	Weight, BMI, %fat, fitness

*Note*: –/–, gender not reported for specific groups.

Abbreviations: %fat, percentage body fat; 1RM, one repetition maximum; 2kmWT, 2‐km walk test; AE‐M, aerobic moderate intensity; AE‐V, aerobic vigorous intensity; BMI, body mass index; BP, blood pressure; COM‐HI, combination of high‐intensity aerobic and high‐load resistance exercise; COM‐LM, combination of low‐moderate intensity aerobic and low‐moderate load resistance exercise; CON, control group, no exercise; FBG, fasting blood glucose; FIT, fitness; HDL, high‐density lipoprotein; HITT, high‐intensity interval training; HR_max_, heart rate maximum; HRR, heart rate reserve; R‐HI, resistance high load; R‐LM, resistance low‐moderate load; RPE, rate of perceived exertion; TG, triglycerides; VO_2_, oxygen uptake; WC, waist circumference.

^a^ Donnelly—same study but results for men and women reported separately.

^b^ Ho—same study but outcomes measures reported in two separate papers.

^c^ Jabbour—same study but outcome measures reported in two separate papers.

^d^ Age range for entire study sample not mean (SD).

In terms of exercise categories, 622 participants (female *n* = 420) were included in the AE‐V category, 1212 participants (female *n* = 1003) in the AE‐M group, 278 participants (female *n* = 247) in HI‐R, 104 participants (female *n* = 45) in R‐LM group, 107 participants (female *n* = 20) in the COM‐HI group and 70 participants (female *n* = 42) in the COM‐LM group. The remaining 1173 participants (female *n* = 919) were controls (no exercise). The mean length of exercise interventions was 20.3 weeks (SD = 13.4), ranging from^17^, ^41^, ^57^, ^58^, ^59^, ^60^, ^70^, ^71^ 8 to 52 weeks,^50^, ^51^, ^54^, ^56^, ^64^ with more than half (59%) of the studies reporting exercise interventions that lasted 12 weeks in duration. The number of weekly sessions and their length was reported in all 45 studies. The average number of sessions per week was 3.2 (SD = 0.54), and the mean length per session was 45.4 (SD = 8.7) min. The majority of studies (82%; *n* = 37) prescribed three sessions of 45‐min exercise per week. Further details relating to the interventions are reported in Table 2.

In relation to outcome measures reported, all but five studies^51^, ^52^, ^53^, ^65^, ^66^ reported BW and similarly all but five reported BMI.^51^, ^61^, ^64^, ^65^, ^73^ Half of the studies (*n* = 23) included WC as an outcome but less than half reported %BF (*n* = 19; 42%). Fitness was measured preintervention and postintervention in 25 of the 45 studies (55.5%). In terms of other metabolic outcomes, TG and HDLs were reported in 16 (36%) studies, FBG in 13 (29%) and systolic/diastolic BP in 12 (27%).

### 3.3 Results of ROB assessment

Details of the ROB assessment in each study included are provided in Table S3. Overall, 13 articles were judged to be of low ROB, # 12 of moderate ROB ** and the remaining 20 rated as at high ROB. †† In terms of each ROB domain, 21 studies showed high or unclear ROB in adequate sequence generation. ‡‡ Nine studies showed low ROB in allocation concealment^17^, ^45^, ^56^, ^59^, ^60^, ^61^, ^67^, ^68^, ^69^ and 13 reported blinding of outcome assessment. §§ Only 12 studies reported an intention‐to‐treat analysis ¶¶ with the remaining 33 studies judged as being either at high or unclear risk of selective reporting. Finally, 14 of the 45 articles included showed high ROB in the domain of risk of other bias. ##

### 3.4 Network meta‐analysis

**FIGURE 2:**
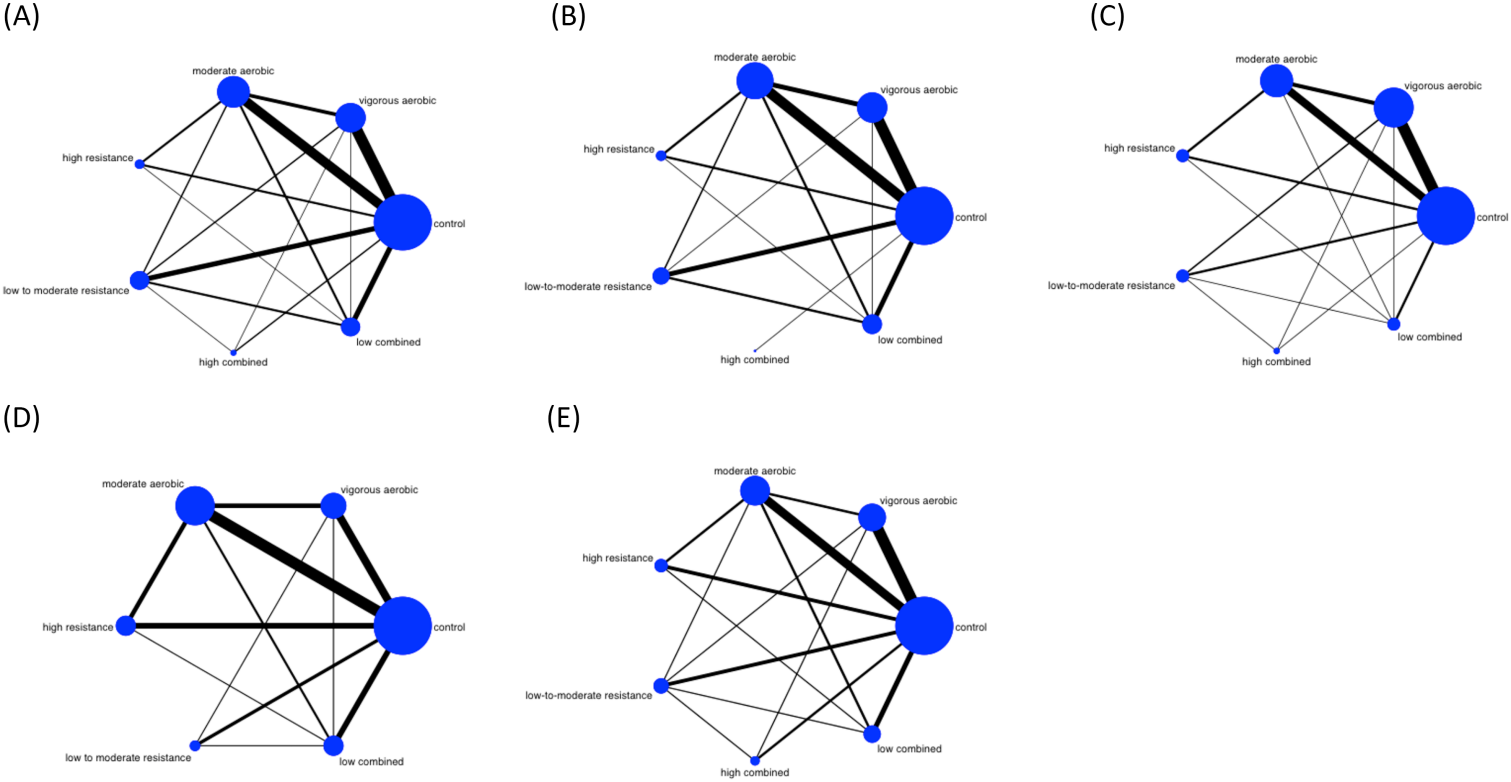
Network meta‐analysis maps of the studies examining the efficacy of exercise intervensions in people living with obesity on (A) weight loss, (B) body mass index, (C) waist circumference, (D) percentage body fat and (E) fitness. The size of the nodes relates to the number of participants in that intervention type, and the thickness of lines between the interventions relates to the number of studies for that comparison

Four measures of anthropometry, BW (kg), BMI, WC (cm) and %BF, and one of CRF (absolute maximal oxygen uptake [L min^−1^]) were included in the NMA. Table S4 details the pre‐post data for all outcomes included in NMA. All networks held the principles of coherency, transitivity and consistency. Due to the small numbers of studies reporting pre‐post intervention levels and/or change in TG, HDL, FBG and BP, it was not possible to include them in a NMA (Table S5). Figure 2 illustrates NMA maps of the studies examining the efficacy of exercise interventions on BW, BMI, WC, %BF and CRF. The size of the nodes relates to the number of participants in that intervention type, and the thickness of lines between interventions relates to the number of studies for that comparison. Further information relating to exercise intervention types contributing to each NMA and the direct and indirect comparisons can be found in Tables S6 and S7. Table 3 details the complete matrix of results, and Table 4 ranks the exercise interventions based on their likelihood of having the desired effect on the outcome being measured.

**TABLE 3:** Network meta‐analysis matrix of results

Outcome	Comparison of treatments: Mean difference (95% confidence intervals) Effect of intervention in each row compared with intervention in each column						
Weight (kg)							
	CON	AE‐V	AE‐M	R‐HI	R‐LM	COM‐HI	COM‐LM
CON		−0.65 (−1.08, −0.17)	−0.75 (−1.24, −0.26)	−0.63 (−1.55, 0.24)	−0.05 (−0.69, 0.59)	−1.01 (−2.71, −0.40)	−0.83 (−2.03, −0.36)
AE‐V			−0.12 (−0.71, 0.47)	−0.03 (−1.01, 0.96)	0.58 (−0.16, 1.32)		−0.38 (−1.14, 0.38)
AE‐M					0.70 (−0.05, 1.46)	−0.08 (−1.36, 1.19)	
R‐HI			*−0.10 (1.00, 0.81)*			−0.18 (−1.67, 1.31)	
R‐LM				*−0.61 (−1.69, 0.47)*			−0.96 (−1.79, −0.14)
COM‐HI		*0.21 (−1.03, 1.44)*			*0.79 (−0.50, 2.07)*		−0.18 (−1.52, 1.17)
COM‐LM			*0.26 (−0.47, 0.99)*	*0.35 (−0.68, 1.39)*			
Body mass index (BMI)							
	CON	AE‐V	AE‐M	R‐HI	R‐LM	COM‐HI	COM‐LM
CON		−0.94 (−1.72, −0.15)	−1.76 (−2.58, −0.95)	−0.93 (−2.48, 0.61)	−0.31 (−1.47, 0.85)	−2.79 (−5.95, −0.36)	−1.56 (−2.71, −0.41)
AE‐V			−0.83 (−1.79, 0.14)		0.63 (−0.71, 1.97)	−1.86 (−5.11, 1.40)	−0.63 (−1.95, 0.70)
AE‐M				0.83 (−0.73, 2.39)		−1.03 (−4.29, 2.23)	0.20 (− 1.06, 1.46)
R‐HI		*−0.01 (−1.69, 1.68)*				−1.86 (−5.37, 1.65)	−0.63 (−2.43, 1.16)
R‐LM			*−1.45 (−2.77,‐0.14)*	*−0.62 (−1.26, 2.51)*			
COM‐HI					*2.48 (0.88, −5.84)*		
COM‐LM					*1.25 (−0.19, 2.07)*	*−1.23 (−4.59, 2.13)*	
Waist circumference (cm)							
	CON	AE‐V	AE‐M	R‐HI	R‐LM	COM‐HI	COM‐LM
CON		−2.03 (−3.55, −0.52)	−2.31 (−3.61, −1.02)	−1.62 (−3.39, 0.15)	−1.43 (−3.75, 0.89)		−2.76 (−4.52, −1.00)
AE‐V			−0.28 (−1.98, 1.42)		0.60 (−2.03, 3.23)		−0.73 (−2.90, 1.44)
AE‐M				0.69 (−1.16, 2.55)			−0.45 (−2.43, 1.53)
R‐HI		*−0.41 (−2.64, 1.81)*					−1.14 (−3.47, 1.19)
R‐LM			*−0.88 (−3.49, 1.72)*	*−0.19 (−3.08, 2.69)*			−1.33 (−4.07, 1.41)
COM‐HI							‐‐
COM‐LM							
Percentage body fat (%)							
	CON	AE‐V	AE‐M	R‐HI	R‐LM	COM‐HI	COM‐LM
CON		−1.27 (−2.59, 0.05)	−1.70 (−3.16, −0.25)	−1.81 (−3.97, 0.35)	−1.47 (−3.60, 0.65)	−2.82 (−5.50, −0.14)	−2.15 (−4.06, −0.25)
AE‐V			−0.43 (−2.24, 1.37)		−0.20 (−2.57, 2.16)		−0.89 (−3.15, 1.38)
AE‐M				−0.11 (−2.41, 2.19)	0.23 (−2.19, 2.65)	−1.12 (−4.12, 1.88)	
R‐HI		*0.54 (−1.94, 3.30)*				−1.01 (−4.43, 2.41)	
R‐LM				*−0.34 (−3.30, 2.63)*		−1.35 (−4.46, 1.77)	−0.68 (−3.34, 1.97)
COM‐HI		1.55 (−1.29, 4.39)					
COM‐LM			*0.45 (−1.70, 2.61)*	*0.35 (−2.30, 2.99)*		*−0.67 (−3.91, 2.58)*	
Cardiorespiratory fitness (VO_2max_, L min^−1^)							
	CON	AE‐V	AE‐M	R‐HI	R‐LM	COM‐HI	COM‐LM
CON		1.29 (−4.66, 2.08)	1.39 (−2.77, 3.37)	1.11 (−8.43, 4.93)	0.14 (−5.55, 5.69)	1.76 (−5.35, 8.87)	0.24 (−0.11.4, 2.93)
AE‐V			1.59 (−2.44, 5.62)		−1.43 (−4.99, 7.84)	3.05 (−4.73, 10.83)	
AE‐M				−2.10 (−8.97, 4.96)			−4.54 (−12.2, 3.14)
R‐HI		*0.42 (−6.37, 7.20)*				3.46 (−6.19, 13.12)	
R‐LM			*0.26 (−5.53, 5.92)*	*1.84 (−10.72, 6.74)*			1.38 (−13.56, 4.70)
COM‐HI			*−1.46 (−8.84, 5.94)*		*−1.62 (−9.86, 6.62)*		
COM‐LM		*2.95 (−4.55, 10.74)*		*−0.54 (−6.17, 5.25)*		*6.00 (−16.07, 4.07)*	

*Note*: Direct comparisons are reported above the grey line whereas indirect comparisons are reported in *italics* below the grey line. Weight, BMI, WC and %BF: clinically desirable outcome is a decrease (−). Cardiorespiratory fitness: clinically desirable outcome is an increase (+).

Abbreviations: AE‐M, aerobic moderate intensity; AE‐V, aerobic vigorous intensity; COM‐HI, combination of high‐intensity aerobic and high‐load resistance exercise; COM‐LM, combination of low‐moderate intensity aerobic and low‐moderate load resistance exercise; R‐HI, resistance high load; R‐LM, resistance low‐moderate load.

**TABLE 4:** Ranking of exercise interventions in order of effectiveness

Weight loss	*P* score^a^	BMI	*P* score	Waist circumference	*P* score	Percentage body fat	*P* score	Fitness	*P* score
COM‐HI	.83	COM‐HI	.85	COM‐LM	.82	COM‐HI	.80	COM‐HI	.73
COM‐LM	.66	AE‐M	.78	AE‐M	.69	COM‐LM	.68	AE‐M	.64
AE‐M	.65	COM‐LM	.69	AE‐V	.59	R‐HI	.58	AE‐V	.39
AE‐V	.55	AE‐V	.45	R‐HI	.45	AE‐M	.55	COM‐LM	.39
R‐HI	.50	R‐HI	.44	R‐LM	.42	AE‐V	.48	R‐HI	.37
R‐LM	.16	R‐LM	.21			R‐LM	.40	R‐LM	.18

Abbreviations: AE‐M, aerobic moderate intensity; AE‐V, aerobic vigorous intensity; COM‐HI, combination of high‐intensity aerobic and high‐load resistance exercise; COM‐LM, combination of low‐moderate intensity aerobic and low‐moderate load resistance exercise; R‐HI, resistance high load; R‐LM, resistance low‐moderate load.

^a^ *P* score ranges from 0 to 1, where 1 indicates best treatment with no uncertainty and 0 indicates worst treatment with no uncertainty.

#### 3.4.1 Body weight

Thirty‐four studies, with 2064 participants and six intervention categories contributed to the NMA assessing BW. Aerobic exercise (AE‐V and AE‐M) contributed 37.2% of the data, resistance training (R‐HI and R‐LM) 12.8%, combined training (COM‐HI and COM‐LM) 11.5% and the control the remaining 38.5% (Table S6). Overall, weight loss was minimal, with mean values ranging from −0.05 to −1.01 kg. Interventions that included an aerobic component (COM‐HI: −1.01 [CI = −2.71, −0.4]; COM‐LM: −0.83 [CI = −2.03, −0.36]; AE‐V: −0.65 [CI = −1.08, −0.17]; and AE‐M: −0.75 [CI = −1.24, −0.26]) were more effective in decreasing BW than control and were superior to resistance only interventions (Table 3). Table 4 illustrates the ranking of the exercise interventions based on their likelihood of being the best or worst for affecting weight loss. COM‐HI had the highest likelihood of achieving weight loss considering both direct and indirect comparison (*P* score = .83).

#### 3.4.2 Body mass index

Twenty‐seven studies, including 1543 participants and all six exercise interventions categories, contributed to this analysis. The majority of the data analysed came from aerobic interventions (39.3%). Resistance and combined training contributed 12.7% and 10.0%, respectively (Table S5). The control group accounted the remainder (38%). All four exercise interventions with an aerobic component (COM‐HI: −2.79 [CI = −5.95, −0.36]; COM‐LM: −1.56 [CI = −2.71, −0.41]; AE‐V: −0.94 [CI = −1.72, −0.15] and AE‐M: −1.76 [CI = −2.58, −0.95]) were found to be significantly more effective in reducing BMI than control and were superior to resistance only interventions. Table 3 illustrates the complete matrix. COM‐HI was the best intervention in the network comparison for decreasing BMI (*P* score = .85) (Table 4).

#### 3.4.3 Waist circumference

Eighteen studies, including 1393 participants and five of the six exercise intervention categories, were included in the NMA for WC. Aerobic exercise interventions contributed over one third of the data to this analysis (35.4%), as did the control group (37.5). The remainder came from resistance (16.7%) and combined (10.4%) training programmes. No study investigated COM‐HI training reported this outcome. While all five exercise interventions reduced WC, COM‐LM (−2.76 [CI = −4.52, −1.00]), AE‐M (−2.31 [CI = −3.61, −1.02]) and AE‐V (−2.03 [CI = −3.55, −0.52]) were superior to resistance interventions and achieved the best results compared with control (Table 2). COM‐LM (*P* score = 0.82) was the best exercise intervention in the network comparison for reducing WC (Tables 3 and 4).

#### 3.4.4 Percentage body fat

Twenty studies, including 1480 participants, reported %BF and were included in the NMA. Over 40% of the data included in the NMA was from the control group (40.1%). Resistance and combined training contributed 12.5% each and aerobic exercise the remaining 34.9%.

The interventions that were found to significantly reduce body fat when compared with control were COM‐HI (−2.82 [CI = −5.50, −0.14]), COM‐LM (−2.15 [CI = −4.06, −0.25]) and AE‐M (−1.70 [CI = −3.16, −0.25]). The exercise intervention with the highest likelihood (*P* score = 0.80) of decreasing %BF was a combination of high‐intensity aerobic and high‐load resistance training (COM‐HI).

#### 3.4.5 Cardiorespiratory fitness

Twenty‐one studies with 1689 participants and all six intervention categories contributed to the NMA assessing CRF. Aerobic exercise interventions contributed the majority of data to this NMA (Table S6), accounting for 45%. Control data contributed 37.2%, resistance training 8.9% and combined the remaining 8.9%. Although all interventions resulted in an increase in absolute VO_2max_, there were no statistically significant improvements in fitness found (Table 3). In accordance with the *P* score, COM‐HI was the intervention in the network comparison most likely to increase VO_2max_ (Table 4).

## 4 DISCUSSION

This NMA represents the most comprehensive analysis of currently available data regarding exercise interventions for people living with obesity. We combined direct and indirect evidence from 45 RCTs comparing six different intervention arms in over 3566 adults classified as having obesity. Our main findings indicate that in subjects with a BMI ≥ 30 kg m^−2^, a combined exercise intervention consisting of aerobic and resistance training (COM‐HI or COM‐LM) is the most promising for reducing WC and %BF, as well as increasing CRF, despite no substantial weight loss. Out of the six interventions investigated, low‐to‐moderate load resistance training was deemed to be the least effective. The p‐score ranking revealed that, when prescribing exercise to improve body composition and fitness, COM‐HI, COM‐LM and AE‐M are the top three exercise intervention for this population. Physiologically, these findings are supported, as while there is some cross‐over between the benefits of aerobic and resistance training, each also contributes specifically to the body's response to exercise. Aerobic training instigates changes in aerobic capacity, improves lipid profiles and increases insulin sensitivity. In addition, particularly in adults with obesity, it can lead to a decrease leptin production, which in turn contributes to a reduction of adipose tissue accumulation. It also increases growth hormone and adiponectin levels, which play a role in lowering abdominal fat and circulating free fatty acids.^78^, ^79^ Resistance training has the potential to change the metabolic properties of skeletal muscles. It results in an increase in lean body mass, lowers exercise‐induced oxidative stress in the overweight and those with obesity and decreases glycated haemoglobin levels in people with an abnormal glucose metabolism.^23^ Hence, it is reasonable to suggest that both types of training together can contribute to greater improvements in both CRF and body composition, and in turn, enhance overall health.

Although weight loss and by association, reduction of BMI, is often the main goal in the treatment of obesity, our results indicate that exercise as an independent intervention, irrespective of which prescription, induced at best, a small reduction in weight loss (mean weight loss ranged from −0.05 to −1.01 kg). Interventions containing aerobic components, whether isolated or as part of a combination training (AE‐V, AE‐M, COM‐HI and COM‐LM), were more effective than resistance training as a single modality. These findings are similar to those reported in previous meta‐analyses exploring the effect of isolated aerobic exercise^19^ and combined exercise interventions^18^ on weight loss, with both authors reporting, that for weight loss, exercise alone is not an effective therapy, a hypocaloric balance is necessary. Hence, dietary intake is key to weight loss and exercise should be combined with diet to optimize its effectiveness for the management of obesity.^80^

Several studies have reported %BF to be more responsive to exercise than BW, and because body fat is the most metabolically harmful tissue type, it may be a more meaningful measure of health change for evaluating exercise interventions.^81^ A recent meta‐analysis reported exercise to have a significantly greater effect on mean change in %BF (−0.39% [CI = −0.5, −0.3]) than on BW (−0.3 kg [CI = −0.5, −0.2]).^82^ However, the effect size reported for both outcomes was small, and as exercise was reported as a single modality irrespective of the mode, frequency or intensity, it is difficult to decipher which specific exercise prescription/s resulted in these changes.^82^ Our NMA goes one step further by providing detailed breakdown by exercise intervention type. We found that the optimal exercise intervention for decreasing %BF is a COM‐HI training programme. This intervention resulted in a much greater reduction in %BF (−2.15 [CI = −4.0%, −0.3%]) than previously reported by Kim et al.^82^ and was shown to be significantly more effective than either isolated aerobic or resistance training.

Beyond total body fat, abdominal adiposity is independently associated with all‐cause mortality and is recognized as a better predictor of obesity‐related conditions than either BW or BMI.^83^, ^84^, ^85^ WC is often used as a surrogate marker of abdominal fat mass, as changes in WC are associated with changes in intra‐abdominal fat and in turn changes in health risk profile.^84^ Findings from a large meta‐regression analysis exploring WC as a predictor of CVD reported an increase of 1 cm in WC resulted in a 2% increase in CVD risk.^84^ Using these findings in reverse and the fact that even modest reductions in WC (<2 cm) confer improvements in all metabolic risk factors,^86^ it is evident from this NMA that COM‐LM exercise was most effective when it came to reducing WC and this is associated with a 5.5% decrease in CVD risk, followed by AE‐V and AE‐M with a 4.5% and 4% decrease, respectively. Furthermore, COM‐LM was more than twice as effective in changing CVD risk compared with either low or moderate load resistance training, a finding that both consistent with^87^ and contradictory^16^ to previously published meta‐analyses.

Until recently, CRF has been overlooked as a potential modifier of the inverse association between obesity and mortality. Despite a growing body of evidence that demonstrates that CRF exerts more influence on morbidity and mortality than %BF and its distribution,^8^, ^88^ even the most recent meta‐analyses investigating the effectiveness of different exercise interventions for adults living with obesity continue to exclusively focus on anthropometric measures to establish efficacy or effectiveness^18^, ^89^ and do not include CRF in their analyses. Others that have included CRF as a primary outcome are not specific to populations with obesity, do not distinguish between exercise types and report fitness in relative terms.

Reporting a change in relative (ml kg^−1^ min^−1^) as opposed to absolute (L min^−1^) VO_2max_ has been shown to hamper interpretation of the data in studies of people with increased body mass.^90^ It can result in overestimated level of fitness as any loss in body mass automatically increases fitness expressed in relative terms, often without the desired underlying metabolic changes.

Although our findings in relation to fitness were statistically insignificant, all exercise interventions brought about an increase in absolute VO_2max_ of between 7% and 15% (Table S4). Previous research has shown an improvement in fitness of between 8% and 10% is associated with a 12% decrease in mortality^6^ and is comparable with a 7‐cm reduction in WC.^7^ When applying this finding to our study, we find that while aerobic exercise (AE‐V = 12.9%; AE‐M = 9.2%) outperformed both types of resistance training (R‐HI = 7.4%; R‐LM = 7.2%) as a single modality, combined low high (COM‐HI = 15%) intensity training resulted in the greatest increase in fitness and consequently should be considered as the preferential exercise intervention to improve CRF in this population.^6^, ^7^

## 5 STRENGTHS AND LIMITATIONS

This study has several strengths and weaknesses. The review was systematic and exhaustive. A considerable sample size of adults living with obesity (*n* = 3566) were included, thus providing the power to detect statistically significant mean differences. Only RCTs, the gold standard for evaluating the effectiveness of an intervention, were included. Incorporating CRF as an outcome measure in the NMA also strengthened this study as it is regularly omitted in obesity studies that evaluate exercise interventions, despite its importance as a predictor and correlate of all‐cause mortality.

Our review shares a number of limitations with the studies on which it is based. Although we attempted to limit heterogeneity by using strict inclusion and exclusion criteria, study populations differed in several respects (age, country of recruitment and ratio of male and female participants). Despite the fact that almost all the included studies prescribed a 3‐day week^−1^, 45‐min session (82%; *n* = 37), there was more variation in the duration of the interventions. However, when included in the analysis as a covariable, intervention duration (number of weeks) did not explain the variance in the effect sizes for any of the outcomes included in the NMA. Results from a previous meta‐analysis reporting the impact of exercise intervention duration^91^ indicates the optimal length differs depending on the outcome being measured and beneficial effectiveness is not indicated for any anthropometric, fitness or metabolic measures until after 8 weeks of continuous training with peak effectiveness being noted anywhere between 12 and 32 weeks. The majority of RCTs in this NMA (82%; *n* = 37), reported exercise interventions ≥ 12 weeks with the average length being 20 weeks (SD = 13.4).

Although the majority of the included trials reported metabolic outcome measures as baseline data (BP, TG, HDL and FBG), they did not report them in postintervention results. Exercise is considered as a cornerstone in the prevention and management of the metabolic syndrome; hence, future trials need to consider these metabolic markers as primary rather than secondary or tertiary outcomes when designing studies.

This NMA identified missing evidence in relation to a number of the exercise categories we included. Aerobic exercise, was, by far, the commonly prescribed intervention, contributing more than half (60–68%) of the intervention data to all five of the NMA's (Table S6). In comparison, resistance training and combined only contributed between 12–18% and 10–18%, respectively. Consequently, due to limited data for head‐to‐head comparisons for some interventions (Table S7), particularly those that compared resistance with combined training, readers should interpret with caution these results as sparse direct evidence can make the analysis less reliable.^92^ This highlights the necessity for further research focusing on resistance and combined exercise interventions in this population.^93^

Finally, 20 of the 45 included RCTs recorded a high risk and 13, a moderate ROB. This remained the case when performance bias was excluded from the global ROB rating, which was high for all studies due to the inability to blind participants to exercise training. Only 12 studies (27%) used an intention‐to‐treat analysis, when reporting their findings, despite this being the gold standard for RCTs. Taken together, more than 70% (*n* = 33) of the included trials were judged as being at moderate to high ROB. Therefore, the results of this present NMA should be interpreted in a conservative manner.

## 6 CONCLUSIONS

Despite its limitations, this NMA provide valuable information for the clinical application of exercise in the management of obesity. In adults with a BMI > 30 kg m^−2^, exercise, as an isolated intervention, irrespective of its prescription components, has a minimal effect on BW. Given the comparatively limited impact on BW and BMI, more focus should be placed on other anthropometric measures such as WC and %BF and perhaps, even more importantly, the measurement of CRF. Based on the most up to date and best available evidence, results from this review suggest the optimal exercise programme to induce the most clinically important changes in WC, %BF and CRF in adults living with obesity is a programme of COM‐HI.

## 7 CLINICAL COMMENT

Going forward, it is imperative that clinicians start to prescribe exercise for adults living with obesity for what it does best: promotion of cardiovascular, respiratory, musculoskeletal and metabolic health. If we continue to promote it as a ‘weight loss’ intervention, it is destined to fail. Emphasizing to clients its importance in reducing %BF and increasing CF, and what this means for their overall health, despite no change in BW, is a move in the right direction, towards optimal exercise prescription.

## CONFLICT OF INTEREST

No conflict of interest was declared.

## TRIAL REGISTRATION

Our study protocol was registered with the International Prospective Register of Systematic Reviews (PROSPERO); registration number: CRD42018111373.

## Supporting information


**File S1:** Search Strategy
**File S2:** Pairwise meta‐analysis (example) for weight loss
**File S3:** Rias of Bias Assessment Results
**File S4:** Pre‐post intervention data for MNA outcomes
**File S5:** Pre‐post intervention data for outcomes not included in the NMA
**File S6:** Exercise categories that contribution to each NMA
**File S7:** Number of studies contributing to direct/indirect analysis for each MNA
**File S8:** Funnel plot graphics

## Footnotes

* References 17, 40, 45, 46, 47, 49, 50, 51, 53, 54, 55, 56, 57, 58, 61, 64, 68.

† References 13, 43, 52, 53, 65, 67, 69, 72, 75, 76, 77.

‡ References 21, 38, 41, 44, 63, 70, 71, 73, 74.

§ References 21, 38, 39, 40, 41, 45, 46, 50, 51, 54, 55, 56, 64, 66, 69, 71, 73, 76.

¶ References 42, 43, 47, 48, 63, 70, 74, 77.

# References 17, 45, 49, 50, 51, 56, 59, 60, 61, 64, 67, 68, 75.

** References 13, 14, 15, 21, 39, 43, 46, 47, 48, 52, 69, 72.

†† References 38, 40, 41, 42, 44, 53, 54, 55, 57, 58, 62, 63, 65, 66, 70, 71, 73, 74, 76, 77.

‡‡ References 21, 39, 40, 41, 42, 44, 47, 48, 51, 54, 55, 57, 58, 62, 63, 65, 66, 70, 71, 74, 77.

§§ References 13, 17, 45, 46, 49, 50, 56, 61, 64, 67, 68, 72, 75.

¶¶ References 17, 45, 48, 49, 51, 56, 59, 60, 61, 64, 67, 68.

## References 13, 38, 40, 42, 44, 46, 48, 54, 63, 65, 66, 70, 71, 77.
