# Supplementary material for: What exercise prescription is optimal to improve body composition and cardiorespiratory fitness in adults living with obesity? A network meta‐analysis
Source: Obes Rev. 2020 Sep 8;22(2):e13137. doi: 10.1111/obr.13137 (PMC7900983; doi:10.1111/obr.13137)
Supplement: Supplementary file 1 — File S1: Search Strategy File S2: Pairwise meta‐analysis (example) for weight loss File S3: Rias of Bias Assessment Results File S4: Pre‐post intervention data for MNA outcomes File S5: Pre‐post intervention data for outcomes not included in the NMA File S6: Exercise categories that contribution to each NMA File S7: Number of studies contributing to direct/indirect analysis for each MNA File S8: Funnel plot graphics [file OBR-22-e13137-s001.pdf]

Supporting Documentation

Supporting file **S1:** Search Strategy

Supporting file **S2:** Pairwise meta-analysis (example) for weight loss

Supporting file **S3:** Rias of Bias Assessment Results

Supporting file **S4**: Pre-post intervention data for MNA outcomes

Supporting file **S5:** Pre-post intervention data for outcomes not included in the NMA

Supporting file **S6:** Exercise categories that contribution to each NMA

Supporting file **S7:** Number of studies contributing to direct/indirect analysis for each MNA

Supporting file **S8:** Funnel plot graphics

Supporting file S1: Search strategy

Database	***Search Terms***		
	Randomised Controlled trial	***Obesity***	***Exercise***
PUBMED	'clinical trials as topic' [MeSH Major Topic] OR (randomized controlled trial) OR (controlled trial) OR (randomized controlled study)	obesity OR overweight	'exercise therapy' [MeSH Major Topic] OR (physical activity) OR exercise OR aerobic OR resistance OR (strength training) OR (combined training) OR (concurrent training) OR (circuit training) OR (HIIT training) OR (interval training)
CENTRAL (Cochrane Central Register of Controlled Trials)	‘controlled clinical trial’ [MeSH term] OR (randomized controlled trial)	obesity OR overweight	‘exercise’ [MeSH term] OR (physical activity) OR aerobic OR resistance OR (strength training) OR (combined training) OR (concurrent training) OR (HIIT training)
EMBASE	‘randomized controlled trial’ [Emtree term] OR (controlled trial) OR (randomized controlled study) OR (clinical trial)	obesity OR overweight	‘exercise’ [Emtree term] OR (physical activity) OR aerobic OR resistance OR (strength training) OR (combined training) OR (concurrent training) OR (fitness training) OR (HIIT training) OR (interval training)
CINAHL and SPORTS DISCUS	(randomized controlled trial) OR (controlled trial) OR (randomized controlled study) OR (clinical trial)	obesity OR overweight	exercise OR (physical activity) OR aerobic OR resistance OR (strength training) OR (combined training) OR (concurrent training) OR (fitness training) OR (HIIT training) OR (interval training)

Supporting file S2: Example of pairwise meta-analysis for weight loss (Intervention duration included as co-variate)

```
replace tx=1 if treat=="control"
replace tx=2 if treat=="Vigorous intensity aerobic"
replace tx=3 if treat=="Moderate intensity aerobic"
replace tx=4 if treat=="Resistance high"
replace tx=5 if treat=="Resistance low"
replace tx=6 if treat=="Combined high"
replace tx=7 if treat=="Combined low"
```

Weight Loss

Control versus aerobic high intensity

```
metan n2 m2 sd2 n1 m1 sd1
```

Study	SMD	[95% Conf.	Interval]	% Weight
2	0.883	0.105	1.662	5.93
3	-2.759	-3.557	-1.962	5.66
6	-2.650	-3.770	-1.531	2.87
9	-0.200	-1.103	0.703	4.41
10	-1.750	-2.724	-0.776	3.79
11	-3.393	-3.977	-2.810	10.57
12	-1.083	-1.380	-0.786	40.82
15	-2.167	-3.272	-1.061	2.95
16	-4.889	-6.284	-3.494	1.85
17	-2.317	-3.370	-1.264	3.24
20	-1.933	-3.147	-0.720	2.44
25	-1.750	-2.749	-0.751	3.60
26	-1.750	-2.800	-0.700	3.26
27	-2.500	-3.317	-1.683	5.39
31	0.167	-0.894	1.227	3.20
I-V pooled SMD	-1.582	-1.772	-1.393	100.00

```
  Heterogeneity chi-squared = 147.71 (d.f. = 14) p = 0.000
  I-squared (variation in SMD attributable to heterogeneity) = 90.5%

  Test of SMD=0: z= 16.35 p = 0.000
```

Control versus treatment 2 (moderate intensity)

```
metan n3 m3 sd3 n1 m1 sd1
```

Study	SMD	[95% Conf.	Interval]	% Weight
1	-0.708	-1.032	-0.385	21.58
5	0.095	-0.434	0.624	8.08
6	-5.417	-6.879	-3.955	1.06
7	0.559	0.336	0.782	45.61
14	-0.083	-0.664	0.497	6.71
15	-1.000	-1.878	-0.122	2.94
17	-1.402	-2.171	-0.634	3.82
22	-2.667	-3.529	-1.804	3.04
24	-3.090	-4.596	-1.583	1.00
26	-2.833	-4.224	-1.442	1.17
28	-6.847	-9.249	-4.445	0.39
30	0.500	-0.460	1.460	2.45
31	0.833	-0.194	1.861	2.14
I-V pooled SMD	-0.178	-0.328	-0.027	100.00

Control versus resistance high

Study	SMD	[95% Conf.	Interval]	% Weight
5	-0.617	-1.154	-0.080	44.94
14	-0.083	-0.659	0.492	39.15
22	-2.917	-3.819	-2.014	15.91
I-V pooled SMD	-0.774	-1.134	-0.414	100.00

Heterogeneity chi-squared = 27.53 (d.f. = 2) p = 0.000
I-squared (variation in SMD attributable to heterogeneity) = 92.7%

Test of SMD=0 : z= 4.22 p = 0.000

Control versus resistance low

Study	SMD	[95% Conf.	Interval]	% Weight
5	-0.617	-1.154	-0.080	44.94
14	-0.083	-0.659	0.492	39.15
22	-2.917	-3.819	-2.014	15.91
I-V pooled SMD	-0.774	-1.134	-0.414	100.00

Heterogeneity chi-squared = 27.53 (d.f. = 2) p = 0.000
I-squared (variation in SMD attributable to heterogeneity) = 92.7%

Test of SMD=0 : z= 4.22 p = 0.000

Combined high versus control

Study	SMD	[95% Conf.	Interval]	% Weight
10	-0.667	-1.509	0.176	65.98
23	-3.417	-4.590	-2.243	34.02
I-V pooled SMD	-1.602	-2.287	-0.918	100.00

Combined low versus control

Study	SMD	[95% Conf.	Interval]	% Weight
4	0.818	-0.058	1.695	14.38
14	-1.333	-1.977	-0.690	26.68
19	-0.250	-0.871	0.371	28.68
24	-3.090	-4.596	-1.583	4.87
25	-1.333	-2.312	-0.355	11.54
28	-7.288	-9.826	-4.751	1.72
29	-4.000	-4.954	-3.046	12.14
I-V pooled SMD	-1.225	-1.557	-0.892	100.00

Heterogeneity chi-squared = 90.81 (d.f. = 6) p = 0.000
I-squared (variation in SMD attributable to heterogeneity) = 93.4%

Test of SMD=0 :z= 7.22 p = 0.000

```
Considerable heterogeneity observed (up to 93.1%) in one or more sub-groups,
Test for heterogeneity between sub-groups likely to be invalid

Significance test(s) of SMD=0

1                     z= 12.21     p = 0.000
0                     z= 10.92     p = 0.000
Overall               z= 16.35     p = 0.000
-------------------------------------------------------------------------

.
TREATMENT CONTROL VERSUS HIGH AEROBIC
 metan n2 m2 sd2 n1 m1 sd1, by(dur_dic) ***DURATION <= 12 weeks= 0 , control versus
high aerobic)

           Study     |     SMD   [95% Conf. Interval]     % Weight
---------------------+---------------------------------------------------
     1> 12 weeks
9                    |   -0.200    -1.103     0.703          4.41
11                   |   -3.393    -3.977    -2.810         10.57
12                   |   -1.083    -1.380    -0.786         40.82
15                   |   -2.167    -3.272    -1.061          2.95
26                   |   -1.750    -2.800    -0.700          3.26
 Sub-total           |
  I-V pooled SMD     |   -1.501    -1.742    -1.260         62.02
---------------------+---------------------------------------------------
     0 <=12 weeks
2                    |    0.883     0.105     1.662          5.93
3                    |   -2.759    -3.557    -1.962          5.66
6                    |   -2.650    -3.770    -1.531          2.87
10                   |   -1.750    -2.724    -0.776          3.79
16                   |   -4.889    -6.284    -3.494          1.85
17                   |   -2.317    -3.370    -1.264          3.24
20                   |   -1.933    -3.147    -0.720          2.44
25                   |   -1.750    -2.749    -0.751          3.60
27                   |   -2.500    -3.317    -1.683          5.39
31                   |    0.167    -0.894     1.227          3.20
 Sub-total           |
  I-V pooled SMD     |   -1.715    -2.023    -1.407         37.98
---------------------+---------------------------------------------------
Overall              |
  I-V pooled SMD     |   -1.582    -1.772    -1.393        100.00
---------------------+---------------------------------------------------

Test(s) of heterogeneity:
               Heterogeneity  degrees of
                 statistic     freedom      P    I-squared**
1                   57.61          4      0.000     93.1%
0                   88.95          9      0.000     89.9%
Overall            147.71         14      0.000     90.5%
Overall Test for heterogeneity between sub-groups:
                    1.15          1      0.283

TREATMENT CONTROL VERSUS HIGH AEROBIC (26 weeks and less versus > 26 weeks)

           Study     |     SMD  [95% Conf. Interval]     % Weight
---------------------+---------------------------------------------------
     0
2                    |    0.883     0.105     1.662          5.93
3                    |   -2.759    -3.557    -1.962          5.66
6                    |   -2.650    -3.770    -1.531          2.87
10                   |   -1.750    -2.724    -0.776          3.79
15                   |   -2.167    -3.272    -1.061          2.95
16                   |   -4.889    -6.284    -3.494          1.85
17                   |   -2.317    -3.370    -1.264          3.24
20                   |   -1.933    -3.147    -0.720          2.44
25                   |   -1.750    -2.749    -0.751          3.60
26                   |   -1.750    -2.800    -0.700          3.26
```

```
27                   |   -2.500    -3.317    -1.683          5.39
31                   |    0.167    -0.894     1.227          3.20
 Sub-total           |
  I-V pooled SMD     |   -1.748    -2.033    -1.462         44.19
---------------------+---------------------------------------------------
     1
9                    |   -0.200    -1.103     0.703          4.41
11                   |   -3.393    -3.977    -2.810         10.57
12                   |   -1.083    -1.380    -0.786         40.82
 Sub-total           |
  I-V pooled SMD     |   -1.451    -1.705    -1.197         55.81
---------------------+---------------------------------------------------
Overall              |
  I-V pooled SMD     |   -1.582    -1.772    -1.393        100.00
---------------------+---------------------------------------------------

Test(s) of heterogeneity:
               Heterogeneity  degrees of
                 statistic     freedom      P    I-squared**
0                  89.54         11      0.000     87.7%
1                  55.86          2      0.000     96.4%
Overall           147.71         14      0.000     90.5%
Overall Test for heterogeneity between sub-groups:
                    2.32          1      0.128

** I-squared: the variation in SMD attributable to heterogeneity)

Considerable heterogeneity observed (up to 96.4%) in one or more sub-groups,
Test for heterogeneity between sub-groups likely to be invalid

Significance test(s) of SMD=0

0                    z= 12.01     p = 0.000
1                    z= 11.20     p = 0.000
Overall              z= 16.35     p = 0.000
```

Body Mass Index

```
. metan _y _stderr, sortby(_y) by(_contrast) random

           Study     |     ES    [95% Conf. Interval]     % Weight
---------------------+---------------------------------------------------
     3 - 1
1                    |   -6.966    -7.809    -6.123          1.60
53                   |   -6.294    -8.023    -4.565          1.22
6                    |   -5.380    -6.708    -4.051          1.40
13                   |   -2.222    -2.806    -1.638          1.69
38                   |   -2.172    -3.315    -1.030          1.48
51                   |   -1.697    -2.767    -0.627          1.51
30                   |   -1.296    -2.040    -0.551          1.64
34                   |   -1.188    -1.846    -0.529          1.66
19                   |   -0.802    -1.394    -0.210          1.68
15                   |   -0.269    -1.108     0.570          1.60
26                   |   -0.204    -1.032     0.624          1.61
62                   |    0.151    -0.831     1.132          1.55
60                   |    0.222    -0.728     1.172          1.56
 Sub-total           |
  D+L pooled ES      |   -2.109    -3.235    -0.983         20.19
---------------------+---------------------------------------------------
     2 - 1
12                   |   -2.564    -3.208    -1.919          1.67
5                    |   -2.092    -3.006    -1.179          1.57
29                   |   -1.413    -2.267    -0.560          1.60
3                    |   -1.412    -2.051    -0.772          1.67
10                   |   -1.376    -1.832    -0.921          1.72
11                   |   -1.087    -1.385    -0.790          1.75
50                   |   -0.990    -1.916    -0.064          1.57
33                   |   -0.467    -1.465     0.530          1.54
```

25	-0.409	-1.279	0.462	1.59
28	-0.365	-1.054	0.324	1.65
44	-0.275	-1.117	0.566	1.60
61	0.000	-1.059	1.059	1.52
9	0.143	-0.759	1.045	1.58
2	0.506	-0.249	1.260	1.63
Sub-total				
D+L pooled ES	-0.878	-1.298	-0.458	22.66
7 - 1				
55	-6.497	-8.268	-4.726	1.20
40	-2.339	-3.506	-1.173	1.47
59	-1.961	-2.631	-1.291	1.66
21	-1.336	-1.948	-0.725	1.68
46	-0.055	-0.932	0.822	1.59
4	0.888	0.001	1.775	1.58
Sub-total				
D+L pooled ES	-1.734	-3.088	-0.380	9.18
3 - 2				
7	-3.288	-4.268	-2.308	1.55
52	-0.707	-1.640	0.226	1.57
31	0.118	-0.576	0.811	1.65
63	0.151	-0.909	1.210	1.51
27	0.204	-0.556	0.965	1.63
14	0.342	-0.052	0.735	1.73
Sub-total				
D+L pooled ES	-0.492	-1.429	0.445	9.65
5 - 1				
54	-4.264	-5.601	-2.927	1.39
32	-1.109	-1.899	-0.319	1.62
39	-0.501	-1.491	0.488	1.54
45	-0.000	-0.856	0.856	1.60
8	0.598	-0.201	1.397	1.62
18	1.049	0.547	1.550	1.71
Sub-total				
D+L pooled ES	-0.622	-1.833	0.589	9.48
4 - 1				
35	-0.871	-1.512	-0.230	1.67
16	-0.576	-1.425	0.273	1.60
20	0.000	-0.575	0.575	1.69
Sub-total				
D+L pooled ES	-0.457	-1.016	0.102	4.96
4 - 3				
17	-0.307	-1.147	0.532	1.60
36	0.317	-0.306	0.939	1.68
22	0.802	0.241	1.363	1.69
Sub-total				
D+L pooled ES	0.334	-0.257	0.926	4.97
7 - 3				
23	-0.535	-1.094	0.025	1.69
57	-0.203	-1.081	0.675	1.59
42	-0.167	-1.148	0.814	1.55
Sub-total				
D+L pooled ES	-0.388	-0.813	0.038	4.83
7 - 4				
24	-1.336	-1.919	-0.754	1.69
Sub-total				
D+L pooled ES	-1.336	-1.919	-0.754	1.69
6 - 1				
37	-2.793	-3.864	-1.722	1.51
Sub-total				

D+L pooled ES	-2.793	-3.864	-1.722	1.51
5 - 3				
41	1.671	0.592	2.750	1.51
56	2.030	1.031	3.030	1.54
Sub-total				
D+L pooled ES	1.864	1.131	2.598	3.05
7 - 5				
58	-2.233	-3.257	-1.210	1.53
43	-1.838	-2.937	-0.739	1.50
49	-0.055	-0.912	0.801	1.60
Sub-total				
D+L pooled ES	-1.347	-2.751	0.056	4.62
5 - 2				
47	0.275	-0.545	1.096	1.61
Sub-total				
D+L pooled ES	0.275	-0.545	1.096	1.61
7 - 2				
48	0.220	-0.620	1.061	1.60
Sub-total				
D+L pooled ES	0.220	-0.620	1.061	1.60
Overall				
D+L pooled ES	-0.956	-1.297	-0.615	100.00

Test(s) of heterogeneity:

	Heterogeneity statistic	degrees of freedom	P	I-squared**	Tau-squared
3 - 1	275.63	12	0.000	95.6%	4.0389
2 - 1	67.35	13	0.000	80.7%	0.4853
7 - 1	70.93	5	0.000	93.0%	2.5838
3 - 2	48.33	5	0.000	89.7%	1.1956
5 - 1	66.50	5	0.000	92.5%	2.0777
4 - 1	4.08	2	0.130	51.0%	0.1241
4 - 3	4.76	2	0.092	58.0%	0.1578
7 - 3	0.63	2	0.730	0.0%	0.0000
7 - 4	0.00	0	.	.%	0.0000
6 - 1	0.00	0	.	.%	0.0000
5 - 3	0.23	1	0.632	0.0%	0.0000
7 - 5	12.07	2	0.002	83.4%	1.2805
5 - 2	0.00	0	.	.%	0.0000
7 - 2	0.00	0	.	.%	0.0000
Overall	804.19	62	0.000	92.3%	1.7050

** I-squared: the variation in ES attributable to heterogeneity)

Supporting file S3: Risk of Bias Assessment

Author Year	Method of Randomisation	Allocation Concealed	Blinding of Participant	Blinding of Research Personnel	Incomplete Outcome data	Dropout Rate %	Other Sources of Bias *	1	2	3	4	5	6	7	Overall Score (Category)
Alizadeh 2013	Block	No	No	Nil stated	No	31	No	L	H	H	H	H	H	H	5 (high)
Alves 2009	Unclear	No	No	Nil stated	Yes	6	Yes	H	H	H	H	L	L	L	3 (moderate)
Arad 2015	Unclear	No	No	Nil stated	No	29	No	H	H	H	H	H	H	H	6 (high)
Arslan 2011	Unclear	No	No	Nil stated	Unclear	not reported	Yes	H	H	H	H	H	H	H	6 (high)
Bonfante 2017	Unclear	No	No	Nil stated	Unclear	not reported	No	H	H	H	H	H	H	H	6 (high)
Borg 2002	Block	No	No	Nil stated	Unclear	24	Yes	L	H	H	H	H	H	L	4 (moderate)
Chih-Hui 2017	Unclear	No	No	Nil stated	No	9	No	H	H	H	H	H	H	H	6 (high)
Church 2007	Block	Yes	No	Yes	Yes	8	Yes	L	L	L	H	L	L	L	0 (low)
Croymans 2014	Block	No	No	Yes	Unclear	<5	No	L	H	L	H	H	H	H	4 (moderate)
Dengel 1996	Unclear	No	No	Nil stated	No	not reported	Yes	H	H	H	H	L	L	H	4 (moderate)

Donges 2013	Unclear	No	No	Nil stated	Yes	0	No	H	H	H	H	L	L	H	4 (moderate)
Donnelly 2013	Stratified	No	No	Yes	Yes	33	Yes	L	H	H	H	H	L	L	2 (low)
Foster-Schubert 2012	Stratified	No	No	Yes	No	9	Yes	L	H	H	H	L	L	L	1 (low)
Frank 2005	Unclear	No	No	Nil stated	Yes	< 5	Yes	H	H	L	H	L	L	L	2 (low)
Gram 2018	Stratified	No	No	Nil stated	No	< 5	Yes	L	H	H	H	H	H	L	4 (moderate)
Herring 2014	Non block	No	No	Nil stated	No	18	Yes	L	H	H	H	H	H	H	5 (high)
Hintze 2018	Unclear	No	No	Nil stated	No	23	No	H	H	H	H	H	H	H	6 (high)
Ho 2012 ±	Sequence	No	No	Nil stated	Unclear	30	Yes	L	H	H	H	H	H	L	4 (moderate)
Ho 2012 ±	Sequence	No	No	Nil stated	Unclear	30	Yes	L	H	H	H	H	H	L	4 (moderate)
Irving 2008	Unclear	No	No	Nil stated	No	25	Yes	H	H	H	H	H	H	L	5 (high)
Irwin 2003	Stratified	Yes	No	Yes	Yes	<5	Yes	L	L	L	H	L	L	L	0 (low)
Jabbour 2015	Unclear	No	No	Nil stated	Unclear	Not reported	Yes	H	H	H	H	H	H	L	5 (high)

Jabbour 2017	Unclear	No	No	Nil stated	Unclear	not reported	Yes	H	H	H	H	H	H	L	5 (high)
Keating 2015	Block	Yes	No	Nil stated	Yes	10	Yes	L	L	H	H	L	L	L	1 (low)
Keating 2017	Block	Yes	No	Nil stated	Yes	<5	Yes	L	L	H	H	L	L	L	1 (low)
Kline 2011	Stratified	Yes	No	Yes	Yes	12	Yes	L	L	L	H	L	L	L	0 (low)
Mengistie 2013	Nil stated	No	No	Nil stated	Unclear	12	Yes	H	H	H	H	H	H	H	6 (high)
Moghasdasi 2012	Nil stated	No	No	Nil stated	Unclear	not reported	No	H	H	H	H	H	H	H	6 (high)
Mohanka 2006	Stratified	No	No	Yes	Yes	<5	Yes	L	H	H	H	L	L	L	2 (low)
Mora-Rodriguesz 2017	Nil stated	No	No	Nil stated	No	15	No	H	H	H	H	H	H	H	6 (high)
Nunes 2016	Nil stated	No	No	Nil stated	No	16	No	H	H	H	H	H	H	H	6 (high)
Quist 2018	Block	Yes	No	Yes	Yes	31	Yes	L	L	L	H	L	L	L	0 (low)
Ross 2015	Stratified	Yes	No	Yes	Yes	28	Yes	L	L	L	H	L	L	L	0 (low)

Rustaden 2017	Block	Yes	No	Nil stated	No	16	Yes	L	L	H	H	H	H	L	3 (moderate)
Sarsan 2006	Nil stated	No	No	Nil stated	No	21	Yes	H	H	H	H	H	L	H	4 (moderate)
Schroeder 2019	Stratified	Yes	No	Yes	No	<5	No	L	L	L	H	L	L	L	0 (low)
Sheikholeslami-Vatani 2015	Nil stated	No	No	Nil stated	Unclear	not reported	No	H	H	H	H	H	H	H	6 (high)
Soori 2017	Nil stated	No	No	Nil stated	Unclear	Not reported	No	H	H	H	H	H	H	H	6 (high)
Stensvold 2010	Block	No	No	Yes	Unclear	not reported	No	L	H	L	H	H	H	H	4 (moderate)
Tjonne 2008	Stratified	No	No	Yes	No	13	Yes	L	H	L	H	H	H	L	3 (moderate)
Tong 2018	Stratified	No	No	Nil stated	No	15	Yes	L	H	H	H	H	H	H	6 (high)
Tseng 2013	Nil stated	No	No	Nil stated	No	9	Yes	H	H	H	H	H	H	H	6 (high)
Tumiati 2008	Block	No	No	Yes	No	31	Yes	L	L	L	H	H	H	L	2 (low)
Van-Aggel Leijssen 2001	Stratified	No	No	Nil stated	Unclear	not reported	Yes	L	H	H	H	H	H	H	5 (high)

Van-Aggel Leijssen 2002	Nil stated	No	No	Nil stated	Unclear	not reported	No	H	H	H	H	H	H	H	6 (high)

± same study

Supporting file S4: Individual study data (pre-post intervention) for all outcome measures included in NMA

Pre-Post Outcomes Measures: mean (SD)

		Body Weight (kg)			Body Mass Index (BMI)			Waist Circumference (cm)			Percentage Body Fat (%)			Fitness (VO_2 max L/min)		
**Authors**	**Exercise category**	**pre**	**post**	**Mean difference (SD)**	**pre**	**post**	**Mean difference (SD)**	**pre**	**post**	**Mean difference (SD)**	**pre**	**post**	**Mean difference (SD)**	**pre**	**post**	**% change**
Alizadeh 2013	CON	84.1 (15.6)		1.8	32.2 (5.3)		0.7									
	AE-M	77.6 (17.3)		1.6	31.6 (4.8)		0.6									
	AE-M	69.1 (15.4)		1.8	29.9 (2.3)		0.7									
Alves 2009	CON	74.5 (1.1)	74.9 (11.3)	0.4 (2.4)	30.3 (3.4)	30.4 (3.5)	0.1 (0.1)									
	AE-M	71.2 (7.8)	69.9 (8.2)	-1.3 (2.4)	29.7 (3.1)	29.1 (3.5)	-0.6 (0.1)									
Arad 2015	CON	85.5 (10.7)	84.4 (10.6)	-1.1 (1.2)	32.1 (3.2)	31.6 (3.6)	-0.5 (1.8)				45.4 (5.0)	44.5 (8.1)	-0.9 (2.5)	1.68 (0.4)	1.76 (0.5)	4.75
	AE-V	90.1 (12.0)	90.2 (14.5)	0.1 (1.5)	32.5 (3.6)	32.7 (3.8)	0.2 (2.1)				54.4 (3.7)	44.5 (4.1)	-9.9 (1.5)	2.08 (0.4)	2.25 (0.5)	8.17
Arslan 2011	CON	84.5 (10.2)	84.8 (10.7)	0.3 (1.0)	32.3 (2.1)	32.4 (2.3)	0.1 (0.7)				39.3 (2.6)	37.6 (2.5)	-1.7 (0.8)			
	AE-V	86.1 (9.6)	83.3 (10.2)	-2.8 (1.2)	34.1 (3.9)	32.9 (3.9)	-1.2 (1.2)				38.8 (3.4)	38.8 (3.4)	0.01 (0.9)			
Bonfante 2017	CON	91.9 (5.5)	90.9 (5.9)	-1.0 (2.2)	30.9 (1.8)	30.5 (1.6)	-0.4 (0.8)	101.4 (4.2)	103.3 (4.9)	1.9 (2.0)	32.7 (5.4)	32.5 (6.3)	-0.2 (2.6)	2.5 (0.4)	2.5 (0.4)	0
	COM-LM															

		93.7	94.5	0.8	30.9	31.2	0.3	102.3	99.6	-2.7	35.3	28.6	-6.7	2.5	2.8	12
		(9.2)	(9.7)	(2.2)	(1.8)	(1.9)	(0.8)	(5.3)	(4.9)	(2.1)	(5.2)	(6.9)	(2.5)	(0.4)	(0.4)	
Borg 2002	CON	92.3	93.9	1.6				98.6	99.5	0.9	33.2	33.4	0.2			
		(10.5)	(11.1)	(2.0)				(7.5)	(8.7)	(2.2)	(1.1)	(1.2)	(0.3)			
	AE-M	91.9	93.7	1.8				98.3	99.1	0.8	34.5	30.1	-4.4			
		(9.3)	(10.7)	(2.2)				(8.3)	(9.1)	(2.3)	(1.8)	(1.6)	(0.5)			
	R-LM	90.8	91.1	0.3				98.1	97.3	-0.8	38.6	35.7	-2.9			
		(8.6)	(8.1)	(2.2)				(8.1)	(8.7)	(2.2)	(2.1)	(1.9)	(0.5)			
Chih-Hui 2017	CON	89.8	90.5	0.7	30.9	30.6	-0.3	98.5	99.3	0.8						
		(4.4)	(4.4)	(1.2)	(0.9)	(0.9)	(0.3)	(2.9)	(2.8)	(1.2)						
	AE-M	92.1	87.2	-4.9	31.8	30.1	-1.7	102.1	95.9	-6.2						
		(3.9)	(3.5)	(1.2)	(0.9)	(0.9)	(0.3)	(2.7)	(2.2)	(1.1)						
	AE-M	84.4	77.7	-6.7	29.6	27.1	-2.5	97.4	88.1	-9.3						
		(2.7)	(2.3)	(1.2)	(0.6)	(0.4)	(0.2)	(2.1)	(1.9)	(0.8)						
	AE-V	87.5	84.6	-2.9	30.7	29.7	-1.1	99.4	94.1	-5.3						
		(4.6)	(4.5)	(1.5)	(0.9)	(0.9)	(0.3)	(2.5)	(2.4)	(1.1)						
Church 2007	CON	85.9	83.7	-2.2				102.6	101.2	-1.4	30.7	29.8	-0.9	1.34	1.30	-2.98
		(12.4)	(11.2)	(2.2)				(11.9)	(9.7)	(1.5)	(5.4)	(5.5)	(0.8)	(0.3)	(0.1)	
	AE-M	85.1	82.9	-2.2				101.5	98.6	-2.9	29.1	28.3	-0.8	1.27	1.36	7.10
		(12.6)	(11.3)	(2.2)				(12.4)	(8.7)	(1.5)	(4.8)	(4.5)	(0.6)	(0.2)	(0.1)	
	AE-M	83.9	83.3	-0.6				99.6	98.2	-1.4	28.6	28.6	0.01	1.35	1.40	3.71
		(11.2)	(11.7)	(2.2)				(11.7)	(9.7)	(1.5)	(4.8)	(4.5)	(0.6)	(0.2)	(0.2)	
	AE-M	83.7	83.3	-0.4				100.1	98.1	-2.0	27.5	28.1	0.6	1.30	1.42	9.23
		(11.3)	(10.3)	(2.2)				(11.1)	(8.5)	(1.1)	(4.1)	(4.4)	(0.5)	(0.2)	(0.2)	
Croymans 2014	CON	98.5	98	-0.5	33.6	33.2	-0.4	106.5	106.7	0.2	26.2	27.9	1.7			
		(14.2)	(15.1)	(1.2)	(3.2)	(3.3)	(2.5)	(7.3)	(9.3)	(4.2)	(6.6)	(8.4)	(3.8)			
	R-HI	96.6	97.1	0.5	30.9	31.1	0.2	103.3	101.4	-1.9	28.6	26.2	-2.4			
		(12.2)	(13.2)	(1.2)	(3.0)	(2.4)	(0.8)	(7.5)	(5.4)	(1.7)	(5.1)	(5.1)	(1.4)			
Dengel 1996	CON	90.1	89.9	-0.2							30.4	30.1	-0.3	2.68	2.61	-2.68
		(4.1)	(4.0)	(1.5)							(1.5)	(1.5)	(0.8)	(0.2)	(0.2)	
	AE-V	94.8	94.3	-0.5							31.0	30.8	-0.2	2.58	2.91	12.79
		(4.8)	(4.9)	(1.5)							(1.2)	(1.6)	(0.6)	(0.19)	(0.18)	
Donges 2013	CON	96.4	95.7	-0.7							23.6	22.2	-1.4	2.07	2.06	-0.48
		(1.7)	(1.7)	(1.2)							(1.4)	(1.5)	(0.6)	(0.2)	(0.2)	
	AE-V															18.94

		96.4	96.6	0.2							27.6	26.8	-0.8	2.31	2.89	
	R-HI	(3.3)	(3.4)	(1.2)							(1.4)	(1.3)	(0.6)	(0.1)	(0.2)	13.41
		103.1	101.1	-2.0							27.8	27 .3	-0.8	1.94	2.20	
	COM-HI	(4.6)	(4.4)	(1.2)							(1.3)	(1.3)	(0.5)	(0.1)	(0.2)	34.83
		92.2	92.3	0.1							23.9	24.3	0.4	2.01	2.71	
		(6.9)	(7.2)	(1.2)							(2.2)	(2.3)	(0.9)	(0.2)	(0.1)	
Donnelly 2013	CON	96.2	96.7	0.5	30.6	30.7	0.1				36.9	35.8	-1.1	3.30	3.10	-6.45
(men)		(11.1)	(12.5)	(1.5)	(4.2)	(4.4)	(3.0)				(4.4)	(5.6)	(2.2)	(0.2)	(0.4)	
	AE-V	102.1	96.2	-5.9	32.1	30.2	-1.9				37 (5.1)	32.5	-4.5	3.72	4.12	10.75
		(11.7)	(14.2)	(1.5)	(3.5)	(4.3)	(1.3)				35.4	(6.8)	(1.6)	(0.5)	(0.7)	
	AE-V	99.9	96.1	-3.8	32.1	30.8	-1.3				(6.8)	32.8	-2.6	3.71	4.21	13.48
		(19.4)	(19)	(1.5)	(5.5)	(5.5)	(2.5)					(7.5)	(2.2)	(0.7)	(0.5)	
Donnelly 2013	CON	78.7	79.2	0.5	29.9	29.1	-0.8				45.1	45.0	-0.1	2.38	2.30	-3.36
(women)		(12.6)	(14.1)	(1.5)	(3.4)	(4.4)	(2.2)				(4.6)	(5.5)	(1.9)	(0.2)	(0.3)	
	AE-V	81.3	76.9	-4.4	29.8	27.5	-2.3				43.5	39.4	-4.1	2.48	2.84	12.01
		(13)	(12.8)	(1.5)	(3.8)	(3.7)	(1.1)				(5.7)	(6.8)	(1.6)	(0.2)	(0.3)	
	AE-V	83.3	79.2	-4.1	30.4	28.9	-1.5				43.6	40.5	-3.1	2.57	2.67	3.95
		(18.9)	(18.1)	(1.5)	(5.6)	(5.4)	(1.9)				(5.8)	(7.2)	(1.7)	(0.4)	(0.4)	
Foster-Schubert	CON	84.2	83.5	-0.7	30.7	30.5	-0.2	94.8	95.7	0.9	47.3	47.1	-0.2	1.93	1.90	-1.55
2012		(12.5)	(12.3)	(1.2)	(3.9)	(4.1)	(0.6)	(10.2)	(9.6)	(1.5)	(4.4)	(5.2)	(0.7)	(0.4)	(0.3)	
	AE-V	83.7	81.7	-2.0	30.7	29.9	0.8	95.1	93.1	-2.0	47.3	45.7	-1.6	1.85	2.02	9.19
		(12.3)	(12.4)	(1.2)	(3.9)	(3.8)	(0.5)	(10.1)	(9.8)	(1.3)	(4.1)	(4.9)	(0.6)	(0.3)	(0.3)	
Frank 2005	CON				30.5						47.4					
					(3.7)						(4.8)					
	AE-M				30.4						47.3					
					(4.1)						(4.1)					
Gram 2018	CON				30.2	30.9	0.7							2.80	2.70	-.3.57
					(2.6)	(3.2)	(1.2)									
	AE-M				29.5	28.9	-0.6							2.70	3.10	14.82
					(1.4)	(1.2)	(0.3)									
	AE-M				30.4	29.8	-0.6							2.70	2.90	7.47
					(3.2)	(3.2)	(0.8)									
	AE-V				30.1	29.3	-0.8							2.70	3.10	14.82
					(1.3)	(2.3)	(0.4)									

Herring 2014	CON				48.6	48.3	-0.3	133.8	132.4	-1.4				3.3	3.4	3.13
					(7.8)	(7.8)	(7.9)	(14.7)	(13.5)	(6.0)				(0.9)	(0.6)	
	R-LM				41.5	40.5	-1	123.7	120	-3.7				4.1	4.30	
					(5.2)	(5.4)	(4.2)	(16.5)	(16)	(6.9)				(0.2)	(0.1)	4.88
	AE-M				44.4	42.6	-1.8	124.9	120.6	-4.3				3.4	3.6	
					(4.4)	(3.9)	(2.7)	(18.2)	(13.3)	(6.8)				(0.3)	(0.3)	5.88
Hintze 2018	CON	79.1	80	0.9	30.1	30.5	0.4									
		(14.1)	(14.3)	(1.2)	(4)	(4.2)	(1.2)									
	R-HI	76.9	78.2	1.3	29.9	31.4	1.5									
		(14.1)	(15.3)	(1.2)	(4.2)	(4.9)	(1.4)									
Ho 2012	CON	85.1	85.1	0	32.4	32.4	0.01	100.3	99.1	-1.2	46.5	46.7	0.2	2.31	2.12	-8.25
		(4.2)	(4.3)	(1.2)	(1.4)	(1.5)	(0.4)	(3.6)	(3.6)	(1.1)	(1.7)	(1.8)	(0.5)	(0.12)	(0.11)	
	AE-M	91.1	91	-0.1	32.7	32.4	-0.3	103.7	101.6	-2.1	44.6	44.1	-0.5	2.26	2.38	6.20
		(4.1)	(4.0)	(1.2)	(1.3)	(1.2)	(0.3)	(2.6)	(2.9)	(0.8)	(1.9)	(1.8)	(0.5)	(0.11)	(0.12)	
	R-ML	89.3	89.2	-0.1	33.0	33 .0	0.01	104.0	101.4	-2.6	43.7	43.2	-0.5	2.21	2.39	8.14
		(4.5)	(4.4)	(1.2)	(1.3)	(1.3)	(0.3)	(3.2)	(3.3)	(0.9)	(1.3)	(1.4)	(0.4)	(0.2)	(0.12)	
	COM-ML	90 .0	88.4	-1.6	33.3	32.8	-0.5	102.2	99.6	-2.6	45.8	44.8	-1.0	2.34	2.49	6.41
		(4.1)	(3.6)	(1.2)	(1.2)	(1.1)	(0.3)	(3.2)	(3.0)	(0.9)	(1.6)	(1.8)	(0.5)	(0.2)	(0.1)	
Irving 2008	CON	89.6	88.7	-0.9	32.7	32.4	-0.3	98.2	97.5	-0.7	45.1	45	-0.1	1.94	1.85	-4.64
		(11.2)	(10.6)	(1.2)	(3.8)	(3.8)	(2.9)	(10)	(8.0)	(4.3)	(3.3)	(3.7)	(1.7)	(0.41)	(0.11)	
	AE-V	97.2	95.1	-2.1	34.7	33.9	-0.8	103.8	102.6	-1.2	44.0	43.6	-0.4	2.03	2.22	9.36
		(22)	(19.3)	(1.2)	(7.5)	(6.5)	(4.8)	(10.6)	(10.4)	(3.8)	(4.9)	(4.1)	(1.6)	(0.41)	(0.4)	
	AE-M	93.5	90.0	-3.5	34.7	33.4	-1.3	103.7	98.1	-5.6	43.5	41.8	-1.7	2.04	2.20	7.84
		(18.3)	(15.6)	(1.2)	(6.8)	(5.6)	(4.6)	(16.8)	(13.3)	(6.2)	(4.8)	(5.4)	(2.1)	(0.32)	(0.12)	
Irwin 2003	CON	81.7		0.7	30.8		0.3				47.4		0.4			
		(12)			(0.8)						(2.1)					
	AE-M	81.6		0.7	30.5		0.3				47.6		0.3			
		(14)			(1.0)						(1.1)					
Jabbour 2015	CON	99.5	100.5	1	33.3	33.7	0.4									
		(24.1)	(21.1)	(0.5)	(4.8)	(3.8)	(2.1)									
	AE-V	101.1	99.9	-1.2	33.2	33.1	-0.1									
		(21.1	(9.1)	(0.5)	(2.8)	(3.7)	(1.5)									
Jabbour 2017	CON	99.5	100.5	1	33.3	33.7	0.4							2.33	2.23	0
		(24.1)	(21.1)	(0.5)	(4.8)	(3.8)	(2.1)							(0.2)	(0.1)	
	AE-V															1.76

		101.1	99.9	-1.2	33.2	33.1	-0.1							2.28	2.32	
		(21.1)	(9.1)	(0.5)	(2.8)	(3.7)	(1.5)							(0.3)	(0.3)	
Keating 2015	CON	90.7	91.5	0.8	32.2	32.9	0.7	93.7	94.5	0.8				1.97	1.95	-1.15
		(4.9)	(5.0)	(0.45)	(1.4)	(1.4)	(2.32)	(1.5)	(1.6)	(0.63)				(0.16)	(0.15)	
	AE-V	103.1	101.8	-1.3	36.3	35.8	-0.5	106.6	104.1	-2.5				2.26	2.53	11.95
		(5.6)	(5.7)	(1.2)	(1.7)	(1.7)	(0.73)	(4.7)	4.5	(1.88)				(0.11)	(0.15)	
	AE-M	88.1	88.9	0.1	31.3	31.5	0.2	95.1	94.1	-1.0				2.39	2.59	8.37
		(3.8)	(3.9)	(1.2)	(0.8)	(1.0)	(0.31)	(2.9)	(2.9)	(1.18)				(0.11)	(0.09)	
		96.1	94.7	-1.4	33.9	33.4	-0.5	104.5	102	-2.5				1.99	2.19	10.52
	AE-M	(5.5)	(18.9)	(1.2)	(0.9)	(0.9)	(0.32)	(2.9)	(3.0)	(1.2)				(0.10)	(0.13)	
Keating 2017	CON	85.5	85.7	3.8	30.8	31.3	0.5	92.3	92.9	0.6				1.84	1.85	0.55
		(3.6)	(3.8)	(0.2)	(1.0)	(1.3)	(0.38)	(2.2)	(2.4)	(0.87)				(0.15)	(0.15)	
	R-HI	86.5	86.6	4.3	32.2	32.2	0.01	97.6	96.3	-1.3				2.04	2.11	3.44
		(4.9)	(4.3)	(0.1)	(1.2)	(1.2)	(0.40)	(3.3)	(3.2)	(1.19)				(0.11)	(0.12)	
Kline 2011	CON	99.3	98.7	-0.6				104.4	105.8	1.4	40.6	40.8	0.2			
		(5.1)	(5.0)	(1.2)				(3.8)	(3.9)	(1.36)	(1.9)	(1.9)	(0.67)			
	COM-LM	105.6	104.7	-0.9				110.8	110.2	-0.6	42.1	41.1	1.1			
		(3.0)	3.3	(1.2)				(2.3)	(2.3)	(0.63)	(1.9)	(1.9)	(0.45)			
Mengistie 2013	CON	86.1		1.0	30.3		0.4	110.5		1.6	37.5		1.1			
		(1.8)		(0.5)	(0.6)		(0.4)	(2.5)		(0.8)	(2.2)		(0.6)			
	COM-LM	86.3		-5.3	30.4		-1.9	111.0		-4.2	37.2		-4.3			
		(3.1)		(1.2)	(0.7)		(0.5)	(2.7)		(1.2)	(3.3)		(1.1)			
Moghasdasi	CON	90.46	90.66	0.2	32.1	32.1	0.01				31.4	31.4	0	2.89	2.89	0
2012		(13.9)	(14.1)	(1.5)	(5.3)	(5.3)	(5.4)				(5.5)	(5.5)	(2.75)	(0.7)	(0.7)	
	AE-V	87.8	85.1	14.1	30.9	29.9	-1.0				29.6	27.3	-2.3	2.82	3.17	12.42
		(8.5)	(8.8)	(0.2)	(2.1)	(2.2)	(1.35)				(3.1)	(3.7)	(1.7)	(0.35)	(0.40)	
Mohanka 2006	CON	81.7												2.64	2.63	-0.38
		(12)												(0.26)	(0.25)	
	AE-M	81.6												2.64	2.97	12.5
		(14)												(0.14)	(0.15)	
Mora-	CON							107	107	0				1.49	1.5	0.67
Rodriguesz								(5.8)	(5.6)	(1.8)				(0.43)	(0.46)	
2017	AE-V	92.6	90.7	-1.9	32.9	31.9		107	105	-2.0				1.4	1.5	7.14

		(13.1)	(11.2)	(2.2)	(8.1)	(8)		(7.3)	(6.3)	(2.15)				(0.5)	(0.41)	
Nunes 2016	CON				32.4			91.5	95.5	4.0	38.2	37.3	-0.9			
					(8.1)			(13.2)	(12.8)	(5.09)	(4.1)	(5.4)	(1.88)			
	R-ML				29.4			88.0	84.0	-4.0	36.9	32.7	-4.2			
					(9.8)			(13.4)	(13.7)	(5.5)	(6.8)	(5.3)	(2.5)			
	R-ML				29.4			90.0	88.0	-2.0	36.8	33.7	-3.1			
					(4.1)			(5.2)	(11)	(3.37)	(3.9)	(3.3)	(1.42)			
Quist 2018	CON	93.2		0.3	30.1									2.55	2.52	-1.18
		(11.8)			(2.6)									(0.8)	(0.7)	
	AE-M	90.7		-0.3	30.4									2.68	2.98	11.19
		(12.7)			(3.1)									(0.5)	(0.6)	
	AE-M	89.5		-0.3	29.2									2.68	2.91	8.58
		(11.1)			(1.9)									(0.5)	(0.6)	
	AE-V	92.4		-0.8	30.1									2.68	3.10	15.67
		(14.1)			(2.4)									(0.6)	(0.7)	
Ross 2015	CON	94.2			33.1			109.5						2.7		
		(17.1)			(4.6)			(10.5)						(0.8)		
	AE-M	95.8			33.5			111.1						2.6		
		(17.9)			(4.9)			(11.2)						(0.7)		
	AE-M	94.0			33.7			110.7						2.7		
		(15.2)			(4.4)			(11.3)						(0.7)		
	AE-V	97.0			33.4			111.3						2.7		
		(16.4)			(4.3)			12.1						(0.7)		
Rustaden 2017	CON	86.4		0.1	30.8		-0.4				30.8		-0.8			
		(14.5)		(0.8)	(5.1)		(0.7)				(6.1)		(1.5)			
	R-HI	86.2		0.3	30.8		-0.3				38.7		-0.8			
		(14.1)		(0.8)	(4.9)		(0.8)				(6.3)		(1.5)			
	R-HI	93.3		0.9	32.2		-0.2				41.1		-1.5			
		(21.1)		(0.2)	(6.1)		(0.8)				(6.2)		(1.7)			
	R-HI	84.4		0.1	30.2		-0.3				38.4		-1.1			
		(14.3)		(0.8)	(5.4)		(0.6)				(6.5)		(2.2)			
Sarsan 2006	CON	86.6	86.4	-0.2	35.4	35.4	0.01	99.7	99.7	0.01				2.18	2.17	-0.45
		(11.3)	(11.5)	(1.2)	(3.7)	(3.7)	(1.5)	(8.2)	(8.7)	(2.7)				(0.4)	(0.3)	
	AE-M	87.5	84.1	-3.4	35.4	33.9	-1.5	99.0	93.2	-5.8				2.38	2.76	15.2
		(11.7)	(12)	(1.2)	(4.9)	(5.0)	(2.4)	(8.7)	(8.7)	(2.7)				(0.5)	(0.4)	

	R-ML	83.8 (9.5)	80.1 (9.5)	-3.7 (1.2)	33.7 (2.9)	32.6 (2.9	-1.1 (1.1)	93.5 (6.4)	93.5 (6.4)	-2.3 (2.0)				2.27 (0.4)	2.49 (0.4)	8.5
Schroeder 2019	CON	91.4 (16)		0.1	32.4 (3.7)		0.0	106.0 (10.0)		0.5	41.4 (5.7)	0.2		2.73 (0.7)		6.22
	AE-V	97.1 (20.7)		-1.0	32.4 (5.2)		-0.3	103.0 (14.0)		0.4	39.1 (8.6)	-0.5		2.89 (0.9)		25.25
	R-HI	93.6 (18.9		-0.2	32.5 (5.9)		-0.1	106 (17.0)		-1.7	39.4 (8)	-0.2		2.91 (0.8)		5.15
	COM-HI	95.8 (21.2)		0.9	31.9 (5.5)		0.2	104 (13.0)		0.9	40.6 (10)	-0.5		3.26 (1.1)		13.19
Sheikholeslami-Vatani 2015	CON	98.5 (5.9)	98.8 (6.1)	0.3 (1.2)	32.1 (1.2)	32.2 (1.3)	0.1 (0.55)				30.9 (3.5)	31.2 (3.7)	0.3 (1.6)			
	COM-HI	97.5 (5.3)	93.5 (4.9)	-4 (1.2)	31.4 (1.1)	30.1 (0.7)	-1.3 (0.39)				28.5 (3.2)	23.6 (2.8)	-4.9 (1.3)			
	COM-HI	96.6 (4.8)	93.1 (3.8)	-3.5 (1.2)	31.9 (0.9)	30.8 (0.7)	-1.1 (0.33)				30.7 (4.3)	26.3 (3.7	-4.4 (1.8)			
Soori 2017	CON	76.5 (2.4)	76.8 (2.5)	0.3 (0.5)	30.7 (1.0)	30.9 (1.1)	0.2 (0.5)				40.1 (1.2)	40.1 (1.1)	0.01 (0.58)			
	AE-M	77.3 (1.3)	74.8 (1.9)	-2.5 (1.2)	31.0 (1.7)	29.9 (1.3)	-1.1 (0.81)				40.2 (2.4)	39.9 (2.4)	-0.3 (1.2)			
	R-ML	76.0 (2.8)	75.8 (2.8)	-0.2 (0.5)	30.1 (1.4)	30 (0.5)	-0.1 (0.47)				40.1 (1.5)	40.1 (1.5)	0.00 (0.75)			
	COM-ML	75.8 (2.7)	73.3 (2.6)	-2.5 (1.1)	30.7 (1.1)	29.5 (0.8)	-1.2 (0.53)				39.2 (1.8)	38.7 (2.0)	-0.5 (0.95)			
Stensvold 2010	CON	100.9 (9.4)	101.6 (10.2)	0.7	31.9 (4.1)	32 (4.2)	0.1	108.7 (9.3)	110.4 (9.0)	1.7				3.4 (1.02)	3.3 (0.9)	-2.94
	AE-V	99.7 (18.7)	98.3 (18.3)	-1.4	31.3 (4.3)	30.9 (4.6)	-0.4	109.6 (10.0)	108.3 (10.7)	-1.3				3.4 (0.91)	3.8 (0.9)	11.76
	R-HI	91.7 (14.7)	92.4 (14.8)	0.7	30.3 (3.5)	30.4 (3.6)	0.1	106.2 (8.6)	105.5 (8.2)	-0.7				2.7 (1.1)	2.9 (0.9)	7.41
	COM-HI	103.4 (17.1)	102.5 (17.2)	-0.9	32.2 (4.2)	32.2 (4.4)	0.01	111.5 (10.8)	110.1 (11)	-1.4				3.3 (0.45)	3.5 (0.5)	6.06
Tjonne 2008	CON	96.4 (12.1)	96.2 (4.9)	-0.2 (1.2)	32.1 (1.1)	32.1 (1.3)	0 (0.6)	114.3 (2.7)	112 (3.4)	-2.3 (1.44)				3.1 (0.33)	3.2 (0.33)	3.22
	AE-M	91.2 (19.5)	87.6 (6.5)	-3.6 (1.2)	29.4 (1.7)	28.2 (1.5)	-1.2 (0.88)	105.1 (5.3)	99.1 (5)	-6.0 (2.57)				3.3 (0.32)	3.7 (0.33)	12.12

	AE-V	91.8 (17.5)	89.5 (4.9)	-2.3 (1.2)	29.8 (1.7)	29.1 (1.5)	-0.7 (0.72)	105.5 (4.1)	100.5 (3.6)	-5.0 (1.64)				3.1 (0.23)	4.1 (0.32)	32.26
Tong 2018	CON	68.2 (9.9)	67.8 (10.4)	-0.4 (1.2)							40.5 (2.6)	40.5 (3.7)	0.01 (0.84)	1.95 (0.19)	1.91 (0.21)	-2.05
	AE-V	67.7 (6.4)	64.9 (6.2)	-1.8 (1.2)							38.4 (2.3)	38.2 (2.4)	-0.2 (0.83)	2.08 (0.28)	2.23 (0.23)	7.22
	AE-V	68.9 (12.1)	64.9 (10.2)	-4.0 (1.2)							38.2 (2.4)	38.2 (2.4)	0.01 (0.84)	2.05 (0.25)	2.50 (0.22)	21.95
Tseng 2013 (1)	CON	95.4 (3.3)	96.2 (3.4)	0.8 (1.5)	31.1 (1.1)	31.3 (1.1)	0.2 (0.43)	99.0 (2.6)	99.6 (2.6)	0.6 (1.16)						
	AE-M	94.3 (4.2)	85.8 (3.7)	-8.5 (1.2)	31.1 (1.1)	28.2 (0.9)	-2.9 (0.43)	101.9 (1.6)	92.6 (1.6)	-9.3 (0.71)						
	R-ML	90.2 (4.2)	84.6 (3.8)	-5.6 (1.2)	30.4 (1.1)	28.5 (1.1)	-1.9 (0.47)	100.8 (2.2)	94.4 (2.0)	-6.4 (0.94)						
	COM-ML	95.7 (4.4)	86.6 (3.8)	-9.1 (1.2)	31.2 (1.2)	28.2 (1.1)	-3 (0,5)	102.6 (2.9)	92.9 (2.6)	-9.7 (1.23)						
Tumiati 2008	CON	102.0 (28)	97.0 (22)	-5.0 (1.5)	39.0 (9.0)	39.0 (8)	0.01 (4.36)	107.0 (14.0)	103.0 (17.0)					3.5 (1.1)	3.3 (1.3)	-5.72
	COM-LM	107.0 (2.07)	96.0 (19.0)	-11.0 (1.5)	38.0 (7.0)	34.0 (9.4)	-4.0 (1.9)	111.0 (16.0)	100.0 (11.0)					3.8 (1.2)	4.6 (1.3)	21.05
Van-Aggel Leijssen 2001	CON	94.7 (14)	94.5 (14.5)	-0.2 (1.2)	32.1 (2.9)	32.4 (3.1)	0.3 (2.47)				44.4 (3.1)	44.8 (3.2)	0.4 (1.81)	2.6 (0.25)	2.5 (0.17)	-3.84
	AE-M	86.5 (10.2)	87.1 (10.1)	0.6 (1.2)	32.8 (3.9)	32.9 (3.6)	0.1 (2.99)				45.0 (4.4)	45.9 (4.3)	0.9 (2.2)	1.9 (0.46)	2.1 (0.41)	10.52
	AE-M	91.2 (9.7)	91.2 (9.3)	0.01 (1.2)	33.3 (3.8)	33.1 (3.9)	-0.22 (4.08)				42.6 (3.1)	42.8 (2.4)	0.2 (0.9)	2.2 (0.19)	2.3 (0.3)	4.53
Van-Aggel Leijssen 2002	CON	96.5 (10.3)	95.9 (9.6)	0.6 (1.2)	31.5 (2.4)	31.4 (2.5)	-0.1 (1.63)				31.3 (4.3)	31.8 (94.)	0.5 (2.5)	2.9 (0.4)	3.03 (0.6)	3.48
	AE-M	102.7 (10.8)	103.1 (11.4)	0.4 (1.2)	31.6 (3.1)	31.7 (3.1)	0.1 (2.3)				31.9 (2.4)	31.5 (2.2)	-0.4 (1.15)	3.2 (0.5)	3.5 (0.5)	9.38
	AE-V	105.5 (6.6)	105.1 (6.2)	-0.4 (1.2)	32.2 (1.6)	32.1 1.3)	-0.1 (1.6)				31.6 (5.1)	31.7 (5.0)	0.1 (2.52)	3.3 (0.4)	3.8 (0.5)	15.15

Supporting file S5: Individual study data (pre-post intervention) for all outcome measures not included in NMA

		Pre-Post Outcomes Measures: mean (SD)														
		**High-density lipoprotein (HDL) mmol/L**			**Triglycerides (TG) mmol/L**			**Fasting blood glucose (FBG) mmol/L**			**Systolic Blood Pressure (SBP) mmHg**			**Diastolic Blood Pressure (DBP) mmHg**		
**Authors**	**Exercise category**	**pre**	**post**	**Mean difference (SD)**	**pre**	**post**	**Mean difference (SD)**	**pre**	**post**	**Mean difference (SD)**	**pre**	**post**	**Mean difference (SD)**	**pre**	**post**	**Mean difference (SD)**
Bonfante 2017	CON	1.12 (0.29)	1.16 (0.19)		2.51 (1.51)	2.49 (1.82)		5.3 (0.5)	5.3 (0.6)							
	COM-LM	1.08 (0.14)	1.06 (0.23)		2.72 (2.19)	2.72 (2.21)		5.2 (0.6)	4.8 (0.6)							
Church 2007	CON	1.50 (0.40)	1.50 (0.30)		1.53 (0.75)	1.52 (0.71)		5.3 (0.6)	5.3 (0.4)							
	AE-M	1.48 (0.31)	1.45 (0.33)		1.51 (0.81)	1.49 (0.68)		5.3 (0.4)	5.2 (0.3)							
	AE-M	1.52 (0.38)	1.45 (0.36)		1.43 (0.65)	1.42 (0.68)		5.2 (0.5)	5.2 (0.6)							
	AE-M	1.41 (0.37)	1.48 (0.31)		1.38 (0.66)	1.38 (0.55)		5.2 (0.5)	5.1 (0.4)							
Croymans 2014	CON										128 (7)	128 (6)		88 (8)	75 (4)	
	R-HI										127 (7)	124 (5)		81 (5)	76 (6)	
Dengel 1996	CON							5.6 (0.2)	5.7 (0.2)							
	AE-V							5.6 (0.2)	5.7 (0.1)							
Frank 2005	CON				1.33 (0.29)	1.34 (0.36)		5.4 (0.5)	5.5 (0.4)							
	AE-M				1.36 (0.22)	1.32 (0.28)		5.6 (0.4)	5.5 (0.4)							

Gram 2018	CON	1.18 (0.30)	1.12 (0.23)	1.10 (0.80)	1.10 (0.60)						
	AE-M	1.31 (0.37)	1.32 (0.36)	1.10 (0.70)	1.20 (0.80)						
	AE-M	1.23 (0.23)	1.30 (0.31)	1.20 (0.70)	1.10 (0.60)						
	AE-V	1.23 (0.29)	1.28 (0.31)	1.30 (0.40)	1.00 (0.50)						
Ho 2012	CON	1.42 (0.11)	1.35 (0.10)	1.25 (0.17)	1.48 (0.23)	5.4 (0.1)	5.36 (0.2)	117 (12)	116 (13)	66 (2)	63 (2)
	AE-M	1.38 (0.09)	1.28 (0.07)	1.36 (0.19)	1.40 (0.16)	5.8 (0.2)	5.7 (0.1)	122 (14)	120 (14)	67 (2)	68 (2)
	R-ML	1.34 (0.08)	1.44 (0.08)	1.27 (0.12)	1.38 (0.18)	5.7 (0.5)	5.8 (0.2)	124 (15)	124 (15)	71 (2)	70 (3)
	COM-ML	1.42 (0.11)	1.41 (0.11)	1.10 (0.10)	1.36 (0.17)	5.7 (0.1)	5.6 (0.1)	115 (13)	113 (14)	66 (2)	64 (2)
Irving 2008	CON	1.18 (0.11)	1.32 (0.23)	2.43 (1.31)	2.71 (1.53)	5.9 (0.8)	6.1 (0.9)	126 (17)	124 (10)	82 (12)	78 (10)
	AE-V	1.14 (0.21)	1.82 (0.11)	2.13 (0.86)	2.21 (1.11)	5.9 (0.8)	5.7 (0.7)	131 (12)	127 (11)	75 (7)	76 (7)
	AE-M	1.21 (0.22)	1.34 (0.20)	1.41 (0.55)	1.43 (0.45)	6 (1.4)	6.3 (1.4)	126 (16)	123 (15)	76 (8)	74 (8)
Keating 2015	CON	1.40 (0.13)	1.40 (0.12)	1.61 (0.32)	1.51 (0.31)	4.0 (0.2)	4.0 (0.2)	119 (4)	119 (13)	78 (2)	76 (8)
	AE-V	1.20 (0,11)	1.30 (0.12)	1.53 (0.21)	1.52 (0.22)	4.5 (0.3)	4.3 (0.3)	123 (5)	123 (4)	83 (2)	80 (2)
	AE-M	1.40 (0.14)	1.30 (0.12)	1.80 (0.31)	1.80 (0.30)	4.3 (0.2)	4.4 (0.2)	125 (5)	123 (4)	77 (3)	76 (3)
	AE-M	1.50 (0.12)	1.40 (0.11)	1.40 (0.20)	1.41 (0.21)	4.4 (0.2)	4.3 (0.1)	116 (4)	116 (2)	79 (2)	73 (3)
Keating 2017	CON	1.80 (0.30)	1.50 (0.20)	1.40 (0.20)	1.40 (0.20)	4 (0.1)	4 (1.2)	118 (4)	117 (2)	78 (2)	76 (2)
	R-HI	1.50 (0.10)	1.70 (0.20)	1.24 (0.10)	1.20 (0.20)	4.1 (0.2)	4.2 (0.2)	117 (3)	117 (2)	77 (2)	76 (2)

Mengistie 2013	CON							5.9 (0.36)		0.2 (0.1)	112 (5)		-3 (2)	81 (4)		2 (1)
	COM-LM							5.7 (0.36)		-0.3 (0.2)	112 (5)		-3 (2)	0 (3)		-2 (1)
Moghasdasi 2012	CON							5.7 (0.47)	5.6 (0.4)							
	AE-V							5.3 (0.4)	4.7 (0.3)							
Mohanka 2006	CON	1.36 (0.40)	1.33 (0.34)		1.51 (0.80)	1.50 (0.90)										
	AE-M	1.34 (0.29)	1.34 (0.31)		1.57 (0.66)	1.46 (0.92)										
Mora-Rodriguesz 2017	CON	0.83 (0.14)	0.86 (0.18)		1.65 (1.20)	1.50 (0.73)										
	AE-V	0.91 (0.18)	0.94 (0.24)		1.54 (0.81)	1.51 (0.92)										
Nunes 2016	CON	1.61 (0.29)	1.57 (0.22)		2.77 (1.10)	2.71 (0.91)										
	R-ML	1.55 (0.20)	1.49 (0.31)		2.98 (1.17)	2.99 (0.91)										
	R-ML	1.72 (0.22)	1.69 (0.30)		2.57 (0.98)	2.54 (0.72)										
Ross 2015	CON	1.21 (0.30)			1.80 (1.0)			5.4 (0.7)			114 (13)		-1 (5)	79 (8)		1 (3)
	AE-M	1.22 (0.30)			1.90 (1.10)			5.4 (0.6)			112 (11)		-5 (8)	80 (8)		-4 (5)
	AE-M	1.20 (0.40)			1.50 (0.81)			5.5 (0.6)			114 (13)		-3 (7)	80 (8)		-2 (4)
	AE-V	1.23 (0.40)			1.60 (0.73)			5.5 (0.5)			114 (16)		-2 (6)	80 (9)		-2 (2)
Sarsan 2006	CON										127 (17)	127 (15)		78 (9)	77 (8)	
	AE-M										121 (20)	120 (20)		80 (10)	73 (9)	
	R-ML													80 (5)	73 (9)	

											117 (12)	117 (13)				
Schroeder 2019	CON	1.39 (0.34)		-0.05	1.37 (0.67)		0.57	5.33 (0.33)			129 (12)		-1	81 (8)		0
	AE-V	1.42 (0.34)		0.00	1.71 (0.77)		-0.28	5.29 (0.4)			131 (10)		0	81 (12)		-2
	R-HI	1.26 (0.33)		0.00	1.88 (1.05)		-0.67	5.67 (0.4)			131 (14)		-1	81 (11)		0
	COM-HI	1.35 (0.38)		-0.05	1.64 (0.38)		0.08	5.44 (0.4)			115 (16)		0	81 (10)		-4
Soori 2017	CON	1.44 (0.19)	1.42 (0.19)		1.36 (0.58)	1.33 (0.54)										
	AE-M	1.32 (0.17)	1.32 (0.15)		1.29 (0.57)	1.21 (0.28)										
	R-ML	1.32 (0.22)	1.39 (0.27)		1.15 (0.22)	1.15 (0.21)										
	COM-ML	1.21 (0.28)	1.43 (0.38)		1.00 (0.31)	0.98 (0.11)										
Stensvold 2010	CON	1.29 (0.35)	1.27 (0.63)		1.81 (0.81)	1.70 (1.00)		6.2 (2.1)	6.1 (2.3)		141 (12)	142 (24)		90 (7)	90 (14)	
	AE-V	1.17 (0.35)	1.23 (0.41)		1.92 (1.00)	1.80 (0.80)		6.0 (1.1)	5.9 (0.8)		136 (15)	134 (12)		89 (8)	85 (6)	
	R-HI	1.38 (0.38)	1.49 (0.64)		2.31 (1.40)	2.30 (1.10)		5.9 (2.4)	5.6 (1.8)		144 (14)	145 (15)		89 (7)	90 (6)	
	COM-HI	1.15 (0.18)	1.23 (0.24)		2.00 (0.90)	1.90 (1.20)		6.1 (2)	6.6 (1.5)		141 (14)	140 (17)		91 (11)	89 (11)	
Tjonne 2008	CON	0.62 (0.05)	0.58 (0.08)								140 (6)	141 (5)		95 (5)	96 (4)	
	AE-M	0.69 (0.07)	0.84 (0.10)								133 (5)	135 (5)		95 (3)	89 (4)	
	AE-V	0.74 (0.09)	0.81 (0.08)								122 (6)	121 (5)		88 (4)	82 (5)	

Tseng 2013	CON	1.21 (0.44)	1.13 (0.40)	4.9 (0.2)	4.8 (0.2)	127 (8)	126 (8)	82 (8)	82 (9)
	AE-M	1.01 (0.21)	0.95 (0.24)	5.0 (0.6)	4.5 (0.4)	120 (10)	120 (10)	76 (10)	76 (10)
	R-ML	1.10 (0.31)	1.05 (0.32)	4.9 (0.9)	4.5 (0.9)	118 (9)	119 (9)	73 (8)	73 (8)
	COM-ML	1.10	1.03	4.8	4.7	123 (9)	123 (9)	78 (10)	78 (10)

Supporting file S6: Additional information relating to exercise intervention type contributing to each network meta-analysis

Weight Loss

Exercise Type	Freq.	Percent	Cum.
control	30	38.46	38.46
vigorous aerobic	16	20.51	58.97
moderate aerobic	13	16.67	75.64
high resistance	3	3.85	79.49
low-to-moderate resistance	7	8.97	88.46
high combined	2	2.56	91.03
low combined	7	8.97	100.00
Total	78	100.00	

Body Mass Index

Exercise Type	Freq.	Percent	Cum.
control	27	38.03	38.03
vigorous aerobic	15	21.13	59.15
moderate aerobic	13	18.31	77.46
high resistance	3	4.23	81.69
low-to-moderate resistance	6	8.45	90.14
high combined	1	1.41	91.55
low combined	6	8.45	100.00
Total	71	100.00	

Waist Circumference

Exercise Type	Freq.	Percent	Cum.
control	18	37.50	37.50
vigorous aerobic	7	14.58	52.08
moderate aerobic	10	20.83	72.92

high resistance	5	10.42	83.33
low to moderate resistance	3	6.25	89.58
low combined	5	10.42	100.00
Total	48	100.00	

Percentage Body Fat

Exercise Type	Freq.	Percent	Cum.
control	20	40.82	40.82
vigorous aerobic	10	20.41	61.22
moderate aerobic	7	14.29	75.51
high resistance	3	6.12	81.63
low to moderate resistance	3	6.12	87.76
high combined	2	4.08	91.84
low combined	4	8.16	100.00
Total	49	100.00	

Fitness

Exercise Type	Freq.	Percent	Cum.
control	21	37.50	37.50
vigorous aerobic	12	21.43	58.93
moderate aerobic	13	23.21	82.14
high resistance	3	5.36	87.50
low-to-moderate resistance	2	3.57	91.07
high combined	3	5.36	96.43
low combined	2	3.57	100.00
Total	56	100.00	

Supporting file S7: Additional information pertaining to number of intervention arms contributing to direct/indirect analysis for each MNA

Weight Loss

Interventions	n=	Percent	Cumulative Percent
AE-V: CON	15	21.74	21.74
AE-M: CON	13	18.84	40.58
AE-V: AE-M	5	7.25	47.83
R-HI: CON	3	4.35	52.17
R-HI: AE-M	3	4.35	56.52
R-LM: CON	7	10.14	66.67
R-LM: AE-V	2	2.90	69.57
R-LM: AE-M	2	2.90	72.46
COM-LM: CON	2	2.90	75.36
COM-LM: AE-V	1	1.45	76.81
COM-LM: R-LM	1	1.45	78.26
COM-HI: CON	7	10.14	88.41
COM-HI: AE-M	1	1.45	89.86
COM-HI: R-HI	3	4.35	94.20
COM HI: R-LM	1	1.45	95.65
COM-HI: COM-LM	3	4.35	100.00

Body Mass Index

Interventions	n=	Percent	Cumulative Percent
AE-V: CON	14	22.22	22.22
AE-M: CON	13	20.63	42.86
AE-V: AE-M	6	9.52	52.38
R-HI: CON	3	4.76	57.14
R-HI: AE-M	3	4.76	61.90
R-LM: CON	6	9.52	71.43
R-LM: AE-V	1	1.59	73.02
COM-HI: CON	2	3.17	76.19
COM-LM: CON	1	1.59	77.78

COM-LM: AE-V	6	9.52	87.30
COM-LM: AE-M	4	6.35	93.65
COM-LM: R-HI	1	1.59	95.24
COM-LM: R-LM	3	4.76	100.00

Waist Circumference

Interventions	n=	Percent	Cumulative Percent
AE-V: CON	7	14.89	14.89
AE-M: CON	10	21.28	36.17
AE-V: AE-M	4	8.51	44.68
R-HI: CON	5	10.64	55.32
R-HI: AE-M	4	8.51	63.83
R-LM: CON	4	8.51	72.34
R-LM: AE-V	1	2.13	74.47
R-LM: AE-M	1	2.13	76.60
COM-LM: CON	5	10.64	87.23
COM-LM: AE-V	1	2.13	89.36
COM-LM: AE-M	2	4.26	93.62
COM-LM: R-HI	1	2.13	95.74
COM-LM: R-LM	2	4.26	100.00

Percentage Body Fat

Interventions	n=	Percent	Cumulative Percent
AE-V: CON	9	21.43	26.19
AE-M: CON	7	16.67	42.86
AE-V: AE-M	2	4.76	47.62
R-HI: CON	3	7.14	54.76
R-HI: AE-M	2	4.76	59.52
R-LM: CON	3	7.14	66.67
R-LM: AE-V	1	2.38	69.05
R-LM: AE-M	1	2.38	71.43

COM-LM: CON	2	4.76	76.19
COM-LM: AE-V	1	2.38	78.57
COM-LM: R-LM	1	2.38	80.95
COM-HI: CON	4	9.52	90.48
COM-HI: AE-M	2	4.76	95.24
COM-HI: R-HI	1	2.38	97.62
COM-HI: R-LM	1	2.38	100.00

Fitness

**Interventions**	**n=**	**Percent**	**Cumulative Percent**
AE-V: CON	10	21.74	21.74
AE-M: CON	12	26.09	47.83
AE-V: AE-M	5	10.89	58.70
R-HI: CON	2	4.35	63.04
R-HI: AE-V	2	4.35	67.39
R-HI: AE-M	1	2.17	69.57
R-LM: AE-V	1	2.38	69.05
R-LM: CON	3	6.52	82.61
COM-HI: CON	2	4.35	86.96
COM-HI: AE-V	1	2.17	89.13
COM-HI: R-HI	1	2.17	91.30
COM-LM: CON	2	4.35	95.65
COM-LM: AE-M	1	2.17	97.82
COM-LM: R-LM	1	2.17	100.00

Supporting File S8: Funnel Plots

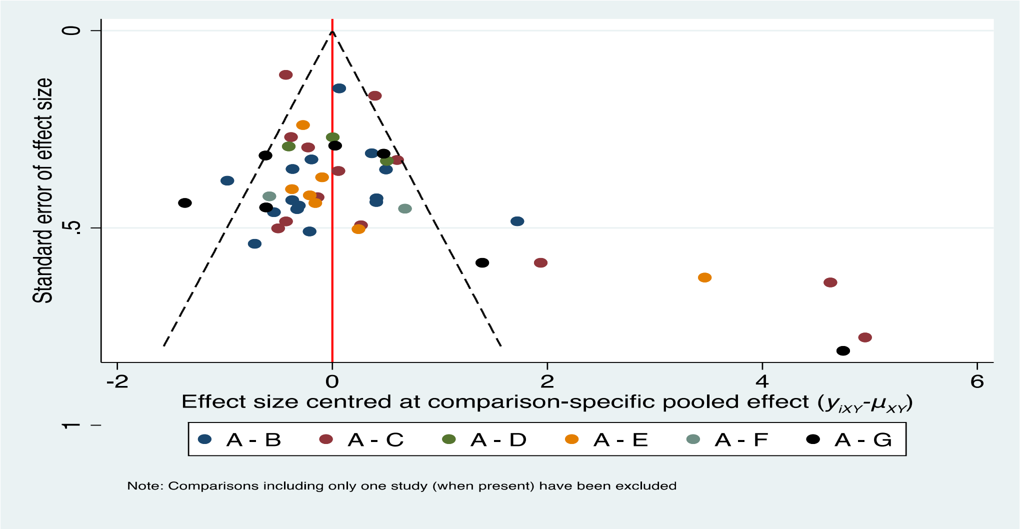

a) Weight loss

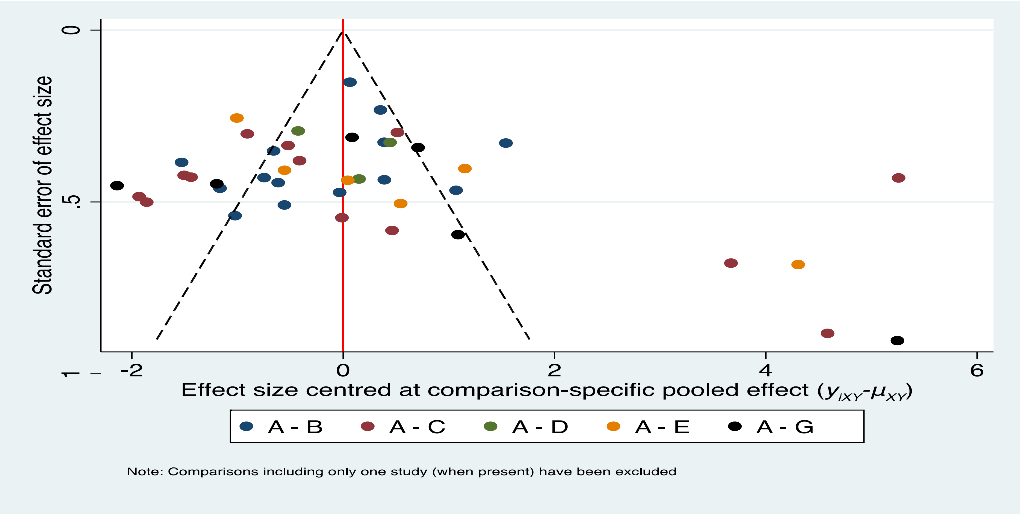

b) Body Mass Index (BMI)

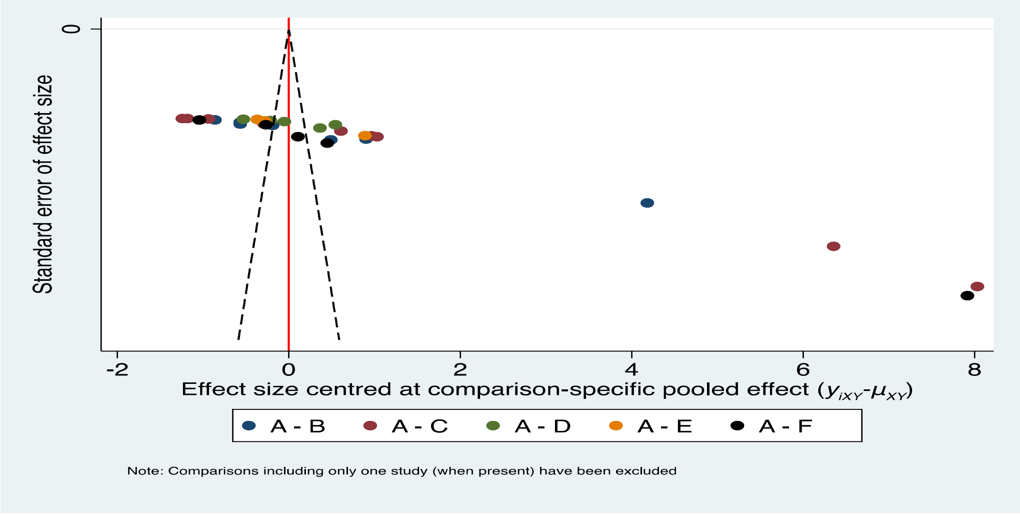

c) Waist circumference (WC)

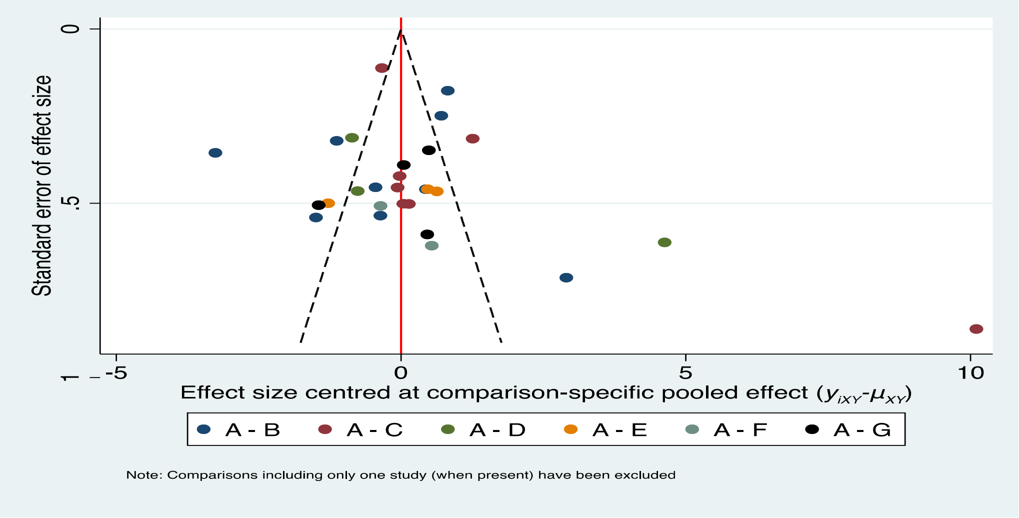

d) Percentage body fat (%)

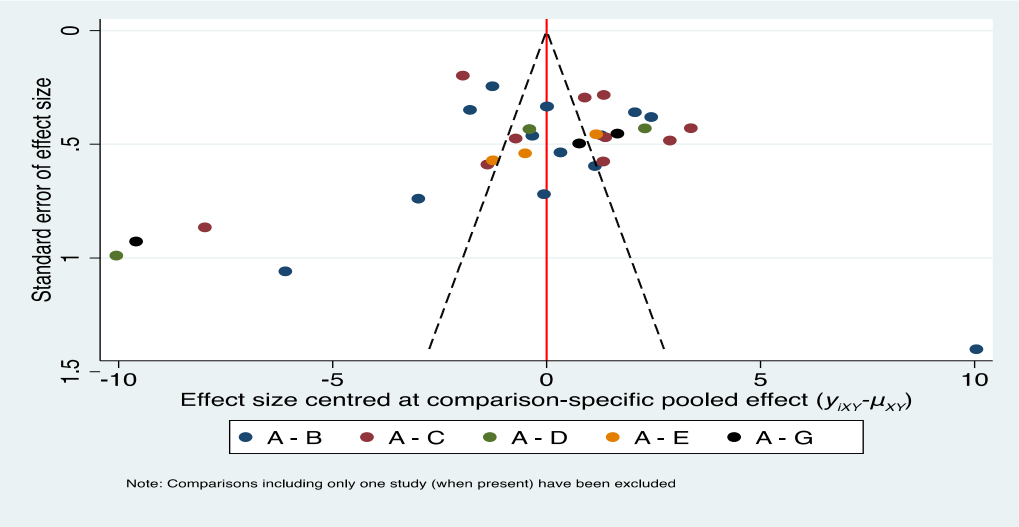

e) Fitness (maximal oxygen uptake)

Figure S8: Funnel plots to illustrate the presence or absence of publication bias in the network meta-analyses. The outer dashed lines indicate the triangular region within which 95% of studies are expected to lie in the absence of both biases and heterogeneity.
